# Comparative transcriptome analysis of juniper branches infected by *Gymnosporangium* spp. highlights their different infection strategies associated with cytokinins

**DOI:** 10.1186/s12864-023-09276-7

**Published:** 2023-04-05

**Authors:** Chenxi Shao, Siqi Tao, Yingmei Liang

**Affiliations:** 1 The Key Laboratory for Silviculture and Conservation of Ministry of Education, College of Forestry, Beijing Forestry University, Beijing, 100083 China; 2 Museum of Beijing Forestry University, Beijing Forestry University, No. 35, Qinghua Eastern Road, Beijing, 100083 China

**Keywords:** RNA-seq, *Gymnosporangium asiaticum*, *Gymnosporangium yamadae*, Gall, Cytokinins, tRNA-isopentenyltransferase, Host-specificity

## Abstract

**Background:**

*Gymnosporangium asiaticum* and *G. yamadae* can share *Juniperus chinensis* as the telial host, but the symptoms are completely different. The infection of *G. yamadae* causes the enlargement of the phloem and cortex of young branches as a gall, but not for *G. asiaticum*, suggesting that different molecular interaction mechanisms exist the two *Gymnosporangium* species with junipers.

**Results:**

Comparative transcriptome analysis was performed to investigate genes regulation of juniper in responses to the infections of *G. asiaticum* and *G. yamadae* at different stages. Functional enrichment analysis showed that genes related to transport, catabolism and transcription pathways were up-regulated, while genes related to energy metabolism and photosynthesis were down-regulated in juniper branch tissues after infection with *G. asiaticum* and *G. yamadae*. The transcript profiling of *G. yamadae*-induced gall tissues revealed that more genes involved in photosynthesis, sugar metabolism, plant hormones and defense-related pathways were up-regulated in the vigorous development stage of gall compared to the initial stage, and were eventually repressed overall. Furthermore, the concentration of cytokinins (CKs) in the galls tissue and the telia of *G. yamadae* was significantly higher than in healthy branch tissues of juniper. As well, tRNA-isopentenyltransferase (tRNA-IPT) was identified in *G. yamadae* with highly expression levels during the gall development stages.

**Conclusions:**

In general, our study provided new insights into the host-specific mechanisms by which *G. asiaticum* and *G. yamadae* differentially utilize CKs and specific adaptations on juniper during their co-evolution.

**Supplementary Information:**

The online version contains supplementary material available at 10.1186/s12864-023-09276-7.

## Background

**Fig. 1:**
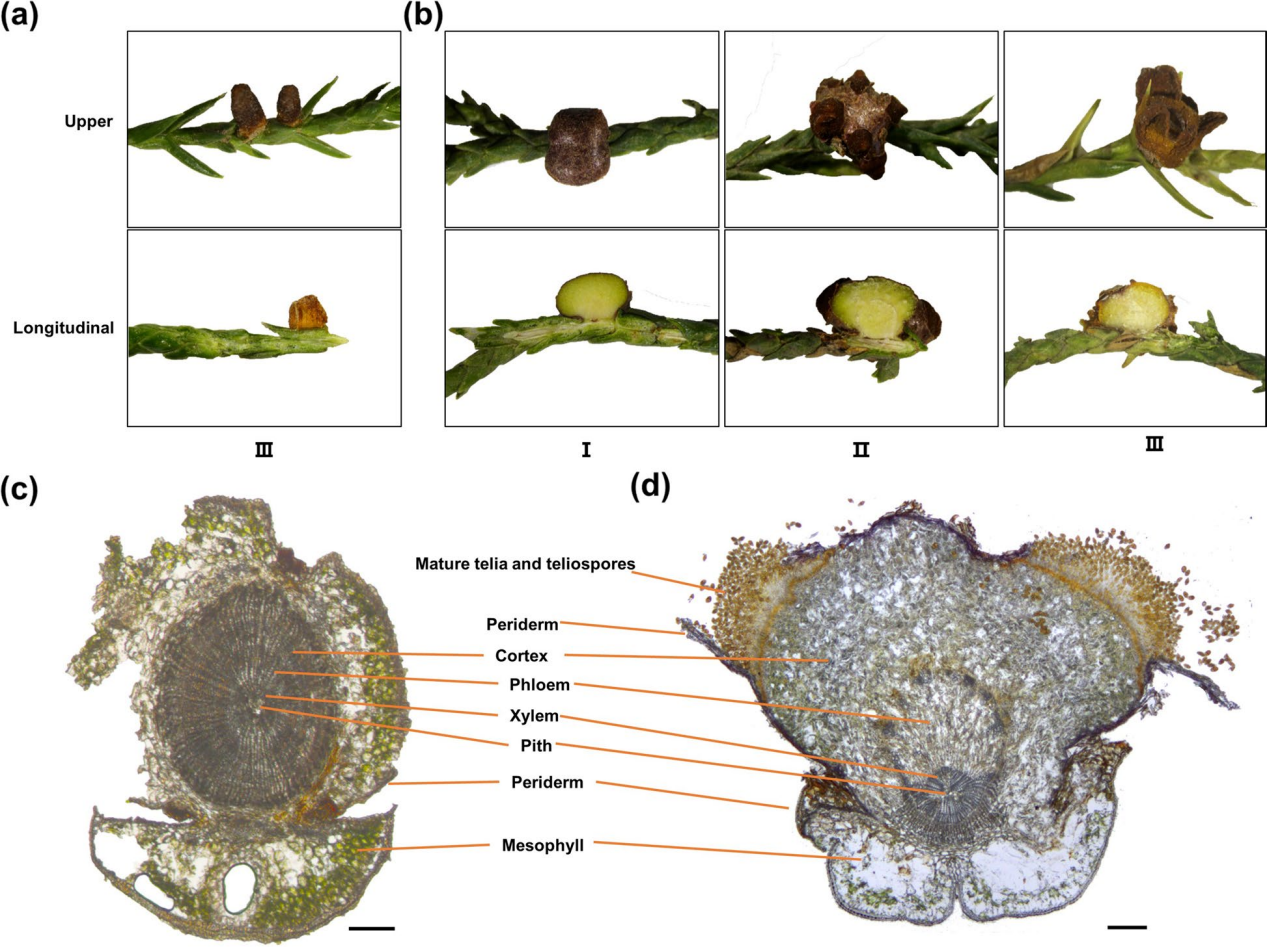
Illustration of symptoms caused by *Gymnosporangium asiaticum* and *G. yamadae* telia on juniper branches. **a** Telia of *G. asiaticum* on juniper leaves and branches. III: the late stage of the teila of *G. asiaticum* with the matured teliospores. **b** Telia of *G. yamadae* on juniper young branches. I: the early stage of the gall development without the *G. yamadae* teliospores formation; II: the middle stage with the *G. yamadae* teliospores break through the gall. III: the late stage of gall development with the mature *G. yamadae* teliospores. **c** Cross-section of healthy juniper young branch. **d** Cross-section of the gall and *G. yamadae* telia on juniper young branch. The *G. yamadae* telia break through periderm with the teliospores spill out and induce the phloem and cortex tissues accreted. Bars: 200 μm

*Gymnosporangium asiaticum* and *G. yamadae* are obligate biotrophic pathogens that cause pear-rust disease and apple-rust disease, respectively, hindering the development of orchard industry [1–3]. In addition, their infections also cause swelling of leaf axil and stems of the important medicinal plant juniper (*Juniperus chinensis*), threatening the cultivation of juniper [4–6]. Both *G*. *asiaticum* and* G*. *yamadae* are demicyclic and heteroecious rust fungi, because they parasite on gymnosperms as telial host, while selecting dicotyledons as aecial hosts [4, 7]. Although they can parasitize on one branch of the juniper at the telial stage, the symptoms are quite different (Fig. 1). Usually, *G. asiaticum* produces tongue-shapes or wedge-shaped telia directly at the base of needle leaves with slightly swelling (Fig. 1a), while *G. yamadae* infection results in galls forming in twigs (Fig. 1b) with enlargement of the phloem and cortex (Fig. 1c, d). After spring rains, the teliospores of *G. asiaticum* germinate preferentially and about a month earlier than *G. yamadae*, then *G. asiaticum* airborne basidiospores infect *Pyrus*, *Chaenomeles* and *Cydonia* species while *G. yamadae* airborne basidiospores infect only *Malus* species without cross-infection [7–9]. Similarly, the mature telia of *G. yamadae* emerge from the galls and germinate successively after several rains (Fig. 1b). The life cycle characteristics of the two *Gymnosporangium* species on junipers suggest a potentially special host selection mechanism, unlike other rust fungi whose telial hosts are angiosperms.

In the last few years, various studies have focused on the host selection mechanisms of heteroecious rust fungi. Related studies on the phylogeny of Pucciniales have demonstrated that the aecial stage is under more selective pressure and stronger evidence of co-diversification with hosts than the telial stage, and the results reflect that the telial hosts are less constrained for Pucciniales species [10–12]. However, phylogenetic analyses did not prove clear host specificity of *Gymnosporangium* species. The overlapping host range of telial stages within *Gymnosporangium* might be driven by multiple speciation mechanisms [8, 13]. Further comparative transcriptomic studies have revealed that stage-specific proteins (e.g., effector proteins and carbohydrate-active enzymes) secreted by heteroecious rust fungi show high correlation with the hosts that probably determine the host-specific selection [14, 15]. Until now, several transcriptome analyses have been performed to investigate the molecular mechanisms underlying the host specificity of *Gymnosporangium*-juniper interaction, from the perspective of rust pathogen. As expected, *Gymnosporangium* species parasitizing on junipers exhibited a conserved genetic expression program corresponding to energy, translation and signal transduction processes [9]. In spite of the composition of CAZymes is similar across different categories, indicating a series of common genes between *G*. *asiaticum* and* G*. *yamadae* that ensure the ability to infect juniper [4], the clearly different symptoms on infected junipers between the two *Gymnosporangium* species imply their potentially different infection strategies.

Evidence showed that pathogens and insect species induce abnormal plant tissues, such as swollen gall, club-shaped root and leaf tumors, providing shelter and nutritional resource for these parasites and symbionts [16–19]. The formation mechanisms of abnormal host plant tissues have been investigated in recent years. Phytohormones, especially indole-3-acetic acid (IAA) and cytokinins (CKs), play key roles in regulating abnormal plant tissue development [20]. Plant parasitic fungi, such as *Taphrina deformans* [21], *Exobasidium gracile* [22], *Moniliophthora perniciosa* [23, 24] and *Ustilago maydis* [19, 25], produce IAA or CKs participating in hyperplasia of host leaf, which contribute to their infections. Certain insect species synthesize IAA and CKs that reprogram and modify plant tissue to a nutrient-rich shelter for them [26–29]. In addition to the effects of parasites-derived phytohormones, other effector molecules including MicroRNAs [30], secreted proteins [28, 31] and small RNAs [32] are deemed to be required to regulate the abnormal growth of host plant. The bottom line is that these organisms that provoke plant tissue proliferation are all dependent on living plants, which may reflect the common survival strategies of these parasites or symbiotic organisms. Rust fungi can induce malformation, swelling and even galls on plant hosts, the symptom is relatively more typical in *Gymnosporangium* species [7, 13, 33, 34]. In *G. juniper*-*virginianae*-cedar pathosystem, the rust fungi synthesized CKs that might facilitate the gall formation [33], which enlighten us postulate that CKs derived by *G. yamadae* might be an important factor that causes galls and results in the different symptoms on juniper between *G. asiaticum*-juniper and *G. yamadae*-juniper pathosystems.

The mechanism of cytokinins biosynthesis is conserved in plants [35]. On the one hand, most isopentenyladenine (iP)- and *trans*-zeatin (tZ)-type cytokinins synthesis are synthesized de novo by the activity of adenylate isopentenyltransferases (IPTs); on the other hand, a large fraction of the cis-zeatin (cZ)-type cytokinins is derived from tRNA degradation, which depends on the catalysis of tRNA-isopentenyltransferase (tRNA-IPTs) [35, 36]. However, all CKs presented in fungi are consistent with origins from within the tRNA degradation pathway [37]. In recent years, investigations of microorganism emphasized the importance of tRNA-IPTs in tRNA degradation pathway of CKs production. The first characterization of tRNA-IPT gene in filamentous fungi was carried out in *Claviceps purpurea* and was proved essential for cZ biosynthesis and contributing to the formation of iP and tZ in the fungus [38]. Research on *Magnaporthe oryzae* [39] and *U. maydis* [25, 37] also demonstrated that tRNA-IPTs mainly contribute to cZ-type CKs production and involve in virulence of these plant pathogens. Several previous studies have also shown that rust fungi, including *Puccinia recondita* f. sp. *tritici* [40] and *G. juniper-virginianae* [33] are able to produce CKs, while the molecular mechanisms of rust-derived CKs as well as their roles in rust-plant interactions remain unclear.

In this study, we executed RNA-seq analysis of juniper branch tissues after the two *Gymnosporangium* spp. infections at different stages and revealed similar and different genetic responses of juniper in the interaction between *G. asiaticum* and *G. yamadae*. The temporal transcriptional analysis of the gall showed an extensive and dynamic gene regulation pattern that is *G. yamadae* dominated. Furthermore, we obtained evidence that *G. yamadae*-derived CKs depended on the catalysis of tRNA-isopentenyltransferase (tRNA-IPT) identified in *G. yamadae* are most likely contribute to galls formation and development, which suggested the distinct infection strategies between *G. asiaticum* and *G. yamadae*. Taken together, the study provides new insights into the host-specificity mechanism of *G. asiaticum* and *G. yamadae*.

## Results

### Experimental design and RNA-seq results

Total RNA for each sample from three independent stages was extracted and subsequently sequenced as a dedicated analysis pipeline (Additional file 1: Fig. S1). Average of 560, 549, 411, and 459 million clean reads per sample were obtained from infected juniper tissues, and 460 and 409 million clean reads per sample from healthy branches for which collected accordingly at the late stage of *G. asiaticum* and *G. yamadae*, respectively (Table 1). The proportion of reads mapped ranged from 87.21% for average in healthy branches sample to 56.65% for average in the late stage of infection branches sample, which reflects the dynamic development of plant tissue and rusts (Table 1).

**Table 1:** Statistics of the RNA-Seq data

Samples	Raw reads	Clean reads	Q20(%)	Q30(%)	Mapping rate(%)
0_GA_J1	49,911,292	48,543,120	98.94	96.35	88.26
0_GA_J2	48,485,060	45,621,494	98.87	96.28	91.77
0_GA_J3	45,523,254	43,857,380	98.92	96.36	91.83
III_GA_J1	54,970,928	53,971,414	98.95	96.46	67.33
III_GA_J2	62,246,294	61,151,994	98.92	96.35	57.30
III_GA_J3	53,866,148	52,939,378	98.93	96.38	62.02
0_GY_J1	41,550,906	40,712,410	98.93	96.31	89.46
0_GY_J2	42,820,094	41,922,276	98.87	96.18	83.41
0_GY_J3	41,353,966	39,997,302	98.93	96.41	88.76
I_GY_J1	53,282,226	52,521,780	98.98	96.50	77.90
I_GY_J2	56,632,488	54,706,312	98.86	96.22	72.28
I_GY_J3	58,615,702	57,415,912	98.87	96.20	75.86
II_GY_J1	42,195,356	40,317,148	98.34	94.85	76.92
II_GY_J2	39,491,260	38,203,854	98.49	95.23	75.52
II_GY_J3	46,570,360	44,720,378	98.49	95.24	73.50
III_GY_J1	43,300,734	40,396,978	98.44	95.33	27.18
III_GY_J2	44,409,450	43,091,794	98.49	95.18	76.29
III_GY_J3	58,332,954	54,177,048	98.49	95.58	66.47

We finally obtained 235,589 unigenes for juniper and 57,513 unigenes for the two *Gymnosporangium* species after separated assembled unigenes between juniper and the two *Gymnosporangium* species (Additional file 2: Table S1). The proportional distribution of homology of *Juniperus chinensis* and the two *Gymnosporangium* species unigenes in NR were shown in Additional file 1: Fig. S2. We assessed the overall reproducibility of the data by combining principal component analysis (PCA) and Pearson correlation analysis, and eliminated III_GY_J2 because of its unclear separation among independent conditions based on gene expression (Additional file 1: Fig. S3). Overall, the transcriptome generated by RNA-seq approach is suitable for subsequent analysis between different samples.

**Fig. 2:**
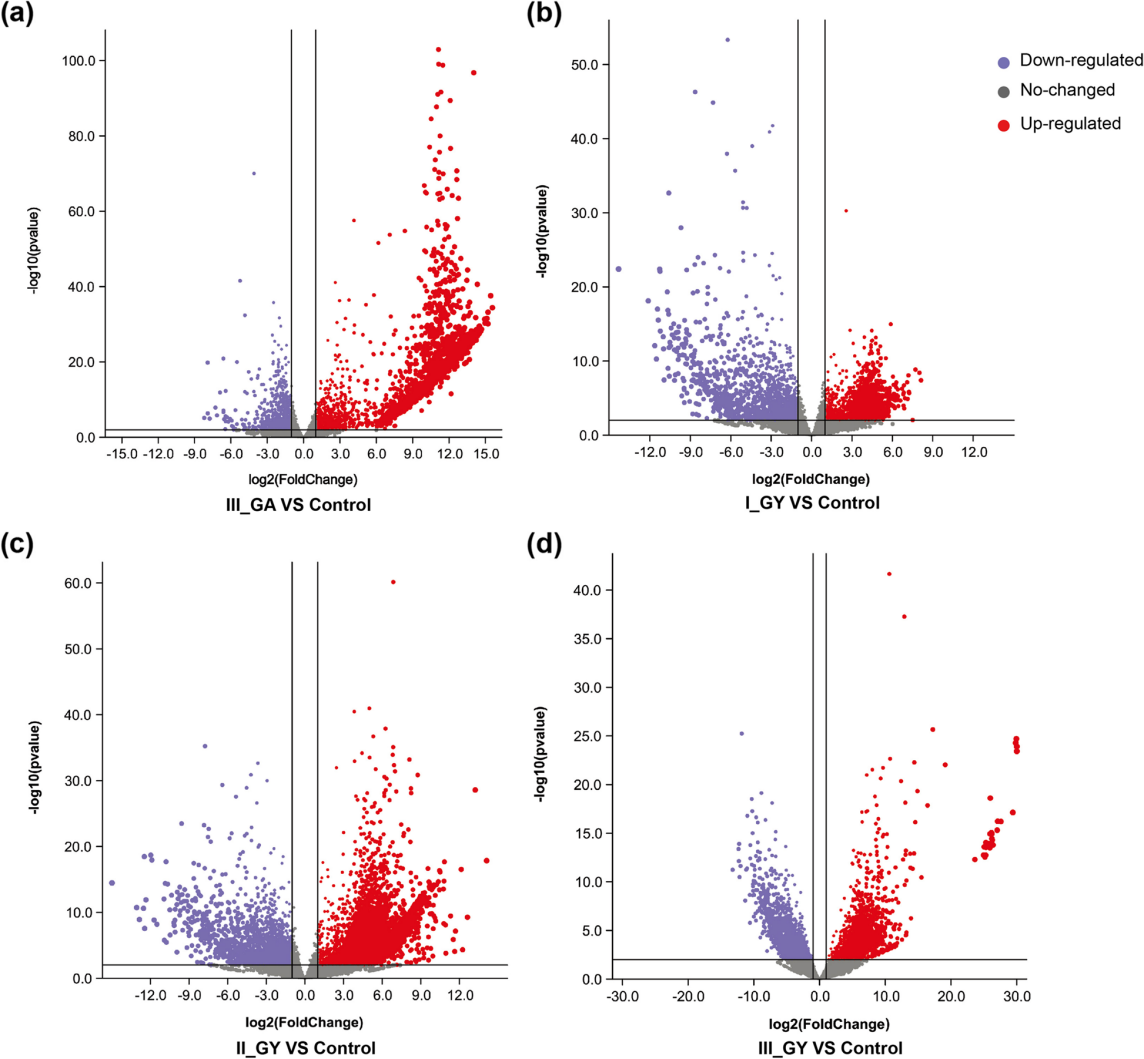
Volcano plots of differentially expressed genes (DEGs). **a-d** DEGs in III_GA_J, I_GY_J, II_GY_J and III_GY_J compared with the control (0_GY_J). The purple dots show down-regulated genes (log_2_Fold-Change <  − 1, *p*-value < 0.05) and the red dots show up-regulated genes (log_2_Fold-Change > 1, *p*-value < 0.05). The y-axis represents − log10 *p*-value, whereas the x-axis represents log_2_Fold-Change

In total, 40,581 and 27,173 CDSs were determined using in juniper and the two *Gymnosporangium* species transcriptomes, respectively. Ultimately, a total of 32,200 (79.35%) unigenes of juniper and 25,623 (94.30%) unigenes of rust were annotated in at least one database, and 9,065 (22.34%) and 6,975 (25.67%) unigenes, respectively, were annotated in all of the above databases (Additional file 2: Table S2). A total of 4,193 differentially expressed genes (DEGs) (3,216 up-regulated and 977 down-regulated) were identified in III_GA_J, likewise 4,843 (2,813 up-regulated and 2,030 down-regulated), 9,522 (7,023 up-regulated and 2,499 down-regulated) and 6,311 DEGs (3,116 up-regulated and 3,195 down-regulated) were identified in I_GY_J, II_GY_J and III_GY_J, respectivly (Fig. 2, Additional file 2: Table S3). These results indicated that the widespread variation in gene expression were induced in the infected plant tissues.

### Transcriptional responses of juniper tissues infected by *G*. *asiaticum* and *G*. *yamadae*

**Fig. 3:**
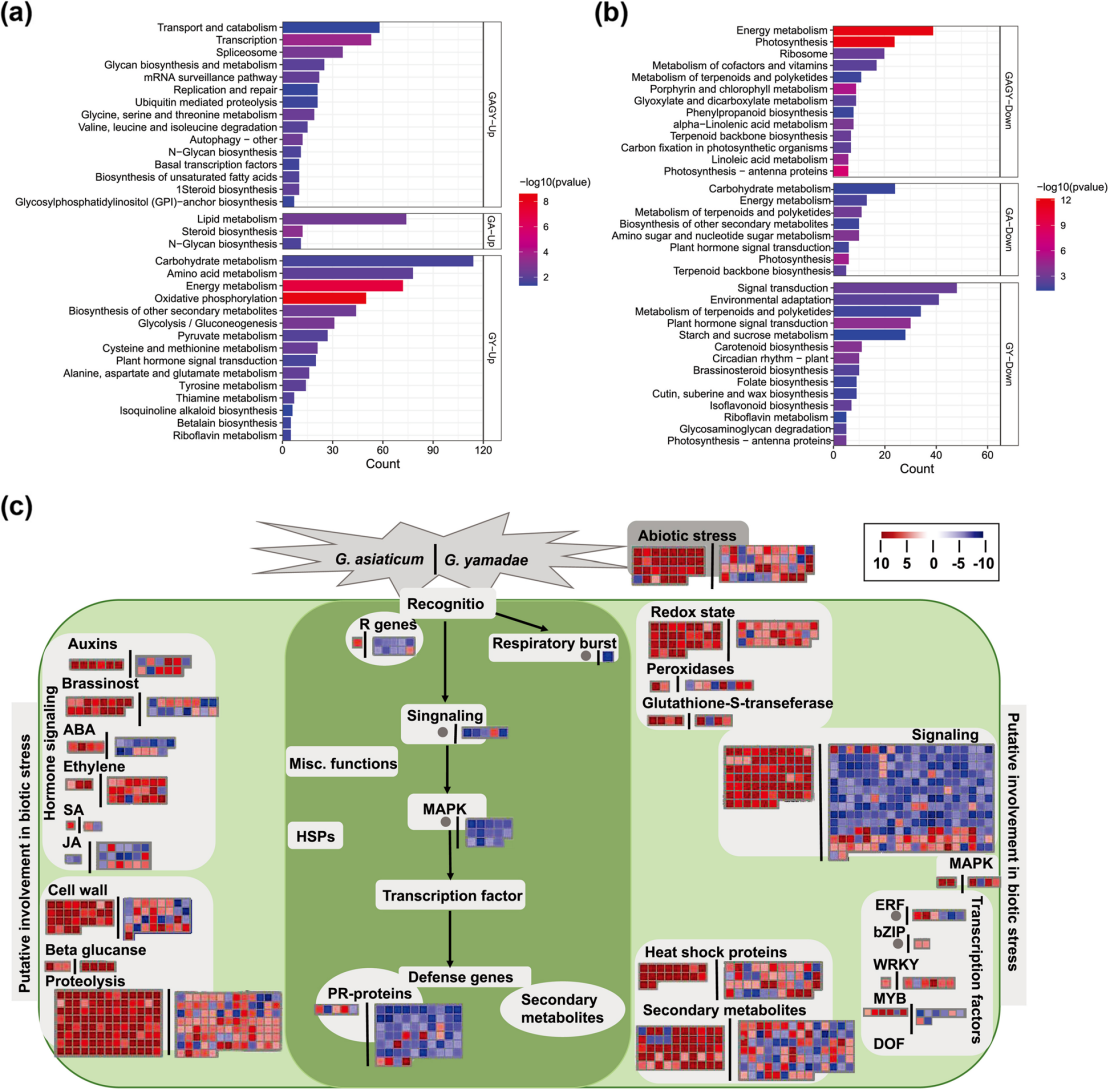
Functional enrichment analysis of differentially expressed genes (DEGs) of juniper infected branch tissues. **a**, **b** KEGG pathway enrichment analysis of DEGs in III_GA_J and III_GY_J. The DEGs with *p*-value < 0.05 and |log_2_Fold-Change|> 1. **c** MapMan biotic stress pathway enrichment of DEGs in III_GA_J and III_GY_J. The DEGs with *p*-value < 0.01 and |log2Fold-Change|≥ 5. III_GA_J on the left side of the black vertical lines, III_GY_J on the right side of the black vertical lines. Blue and red squares were denoted increased (positive) and decreased (negative) DEGs; gray circles indicate no hit

The Venn diagram (Additional file 1: Fig. S4) showed that 1,391 DEGs were commonly up-regulated and 580 DEGs were commonly down-regulated in III_GA_J sample and III_GY_J sample. KEGG pathway enrichment analysis of these DEGs (Fig. 3) showed that the commonly up-regulated genes were mostly enriched in ‘transport and catabolism’, ‘transcription’, ‘spliceosome’ pathways. Conversely, the commonly down-regulated genes were predominantly enriched in ‘energy metabolism’, ‘photosynthesis’, and ‘ribosome’ pathways (Fig. 3a). Furthermore, the DEGs enriched in the 'carbohydrate metabolism', 'enrgy metabolism' and 'biosynthesis of other secondary metabolites' pathways were up-regulated in III_GY_J sample but down-regulated in III_GA_J sample (Fig. 3b).

We noted that there 27 DEGs (Type_a) were downregulated in III_GA_J sample while upregulated in III_GY_J sample, 226 DEGs (Type_b) were upregulated in III_GA_J sample while downregulated in III_GY_J sample (Additional file 1: Fig. S4). Based on Swissport annotations, half of DEGs in Type_a relate to plant immunity, such as berberine bridge enzyme-like 18 and probable aspartic proteinase GIP2; some genes relate to plant hormone synthesis and cell growth and development, such as 1-aminocyclopropane-1-carboxylate oxidase, 12-oxophytodienoate reductase 3, protein SOSEKI 1 and protein ASMMETRIC LEAVES 2 (Additional file 2: Table S4). In Type_b DEGs, most genes were related to plant immune defense, such as leucine-rich repeat receptor-like serine/threonine-protein kinase, cysteine-rich receptor-like protein kinase 10 and aspartic proteinase nepenthesin-2 (Additional file 2: Table S4). However, plant defense-related genes were differently regulated in juniper after infection by *G. asiaticum* and *G. yamadae*.

MapMan analysis (Fig. 3c) showed that a large number of genes were significantly regulated in signaling, proteolysis and secondary metabolism categories during *G. asiaticum* and *G. yamadae* treatments. However, *G. yamadae* infection inhibited transcription of the cascades associated with the plant defense mechanism after recognition by the corresponding receptors, while fewer DEGs identified during *G. asiaticum* infection (Fig. 3c, Additional file 2: Table S5). More importantly, most *G. yamadae* DEGs were downregulated, especially genes involved in signaling (262 DEGs) and PR-proteins (64 DEGs), compared to *G. asiaticum* DEGs (66 and 5 DEGs in signaling and PR-proteins) (Fig. 3c, Additional file 2: Table S5). Hormone signaling-related genes, including ethylene, auxins, ABA and JA were downregulated in III_GY_J sample while highly expressed III_GA_J sample (Fig. 3c).

### *G. yamadae* induces widespread transcriptomic changes in galls

**Fig. 4:**
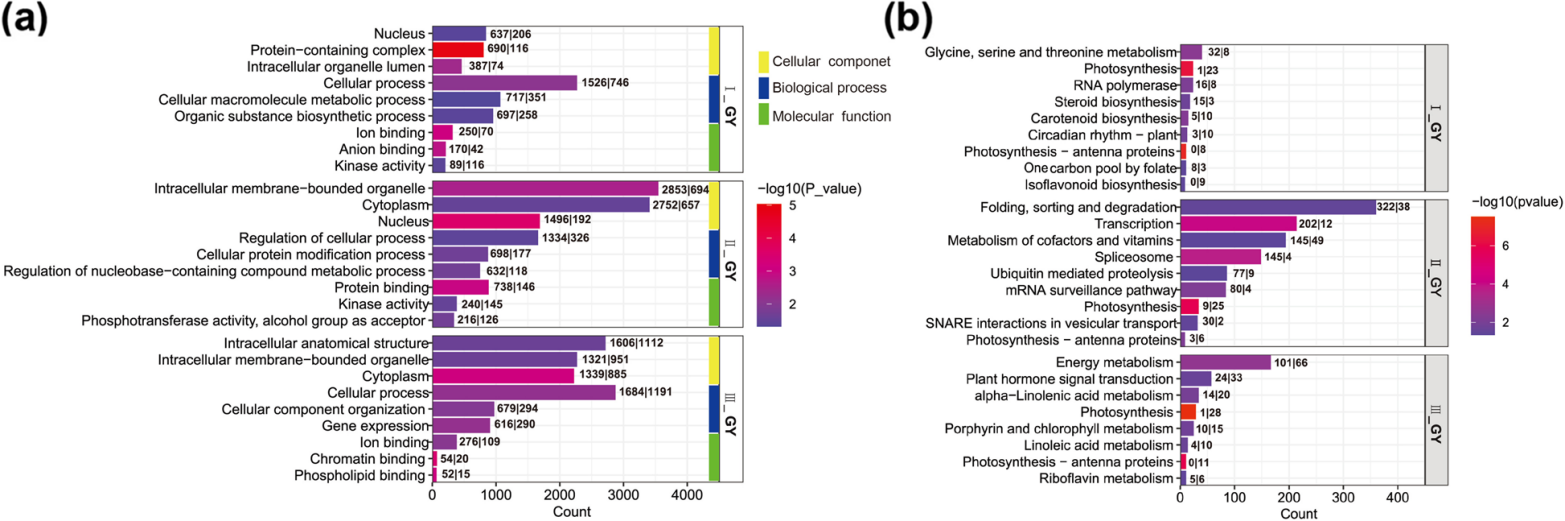
GO and KEGG pathway enrichment for differentially expressed genes (DEGs) in the gall development stages. **a**, **b** GO and KEGG pathway enrichment analysis of DEGs of juniper branches infected by *Gymnosporangium yamadae*. The DEGs with a *p*-value < 0.05 and |log2Fold-Change|> 1. I_GY_J: the early stage of the gall development without the teliospores formation; II_GY_J: the middle stage with the teliospores break through the gall; III_GY_J: the late stage of gall development with the mature teliospores. The numbers on the left and right sides of the vertical bar at the top of the bar chart represent the up-regulated and down-regulated genes enriched to the term, respectively

The functional enrichment analyses in GO databases showed that mainly up-regulated genes were mostly classified into cellular component and biological process categories. The common GO terms, including ‘nucleus’ and ‘kinase activity’ between I_GY_J and II_GY_J; ‘cytoplasm’ and ‘intracellular membrane-bounded organelle’ between II_GY_J and III_GY_J; ‘cellular process’ and ‘ion binding’ between I_GY_J and III_GY_J (Fig. 4a). KEGG pathways analysis indicated that DEGs were commonly significantly enriched in the photosynthesis-related pathways, with most of them being down-regulated across all three stages. The rest of the enriched pathways, however, varied among the three stages. In I_GY_J sample, pathways such as ‘glycine, serine and threonine metabolism’, ‘RNA polymerase’, ‘steroid biosynthesis’ were significantly enriched; in II_GY_J sample, pathways representing ‘folding, sorting and degradation’, ‘transcription’ and ‘metabolism of cofactors and vitamins’ were significantly enriched; in III_GY_J sample, ‘energy metabolism’, ‘plant hormone signal transduction’ and ‘alpha-linolenic acid metabolism’ pathways were predominantly enriched, which mainly involved with up-regulated genes (Fig. 4b).

**Fig. 5:**
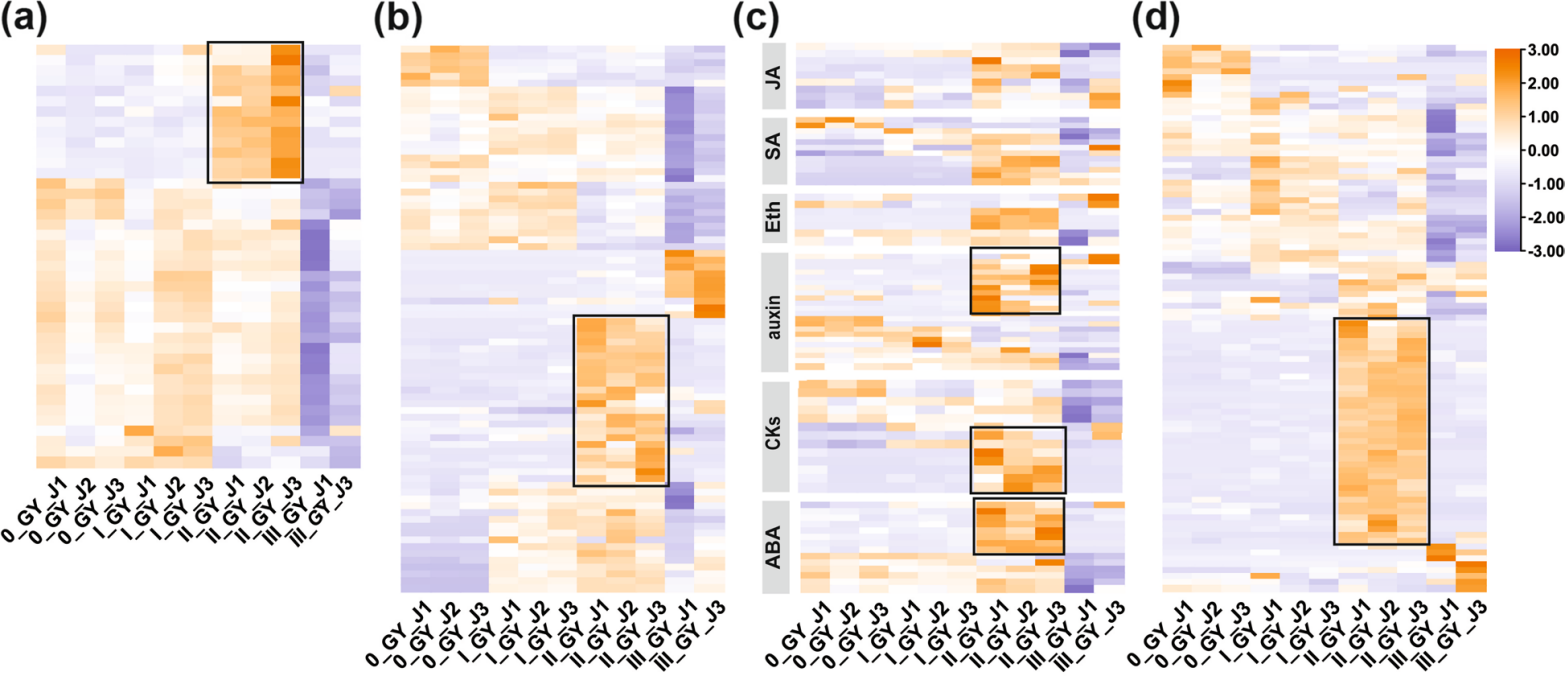
Gene expression changes for photosynthesis, sugar metabolism, plant hormone and defense. **a-d** Heatmaps of differentially expressed genes related to photosynthesis, sugar metabolism, plant hormone and plant defense pathways. The differentially expressed genes with a *p*-value < 0.05 and |log2Fold-Change|> 1. Expression is labelled from lowest (blue) to highest (orange) in each row. Expression is labelled from lowest (purple) to highest (orange) in each row. Black boxes highlight the patterns of II_GY_J sample discussed in the text. JA: jasmonic acid, SA: salicylic acid, Eth: ethylene, CKs: cytokinins, ABA: abscisic acid

The heatmaps showed that during the middle stage (II_GY_J) of gall development, genes associated with photosynthesis, sugar metabolism, plant hormones and defense-related pathways were predominantly up-regulated at (Fig. 5, Additional file 2: Table S6). The most significant up-regulated genes in II_GY_J sample involved in photosynthesis are the light-harvesting chlorophyll protein complexes LHCB4, psaG in photosystem I, cytochrome b6f complex petC, psbW in photosystem II and photosynthetic electron transport petF (Fig. 5a, Additional file 2: Table S6). Additionally, there is another notable cluster of up-regulated genes in sugar metabolism pathway, including beta-glucosidase (which demonstrated a 13-fold increase), and glycogen synthase and trehalose 6-phosphate synthase (both of which had an eightfold increasein the II_GY_J sample) (Fig. 5b, Additional file 2: Table S6). Genes involved in jasmonic acid (JA), salicylic acid (SA) and ethylene (Eth) signal transduction pathways showed a common expression pattern, which were up-regulated gradually from the early stage to the middle stage and repressed at the late stage of the gall development (Fig. 5c). In contrast, a set of genes in cytokinins (CKs), abscisic acid (ABA) and auxin signal transduction pathways were particularly up regulated II_GY_J sample, such as auxin influx carrier, cytokinins receptor and ABA receptor (Fig. 5c, Additional file 2: Table S6). Plant defense-related genes were extensively up-regulated in II_GY_J sample, especially some typical plant immune signal recognition receptors such as calcium-dependent protein kinase and calmodulin, as well as some bacterial induced defense genes (Fig. 5d, Additional file 2: Table S6).

### Identification of CKs biosynthesis-related gene *tRNA-IPT* in *G*.* yamadae*

**Fig. 6:**
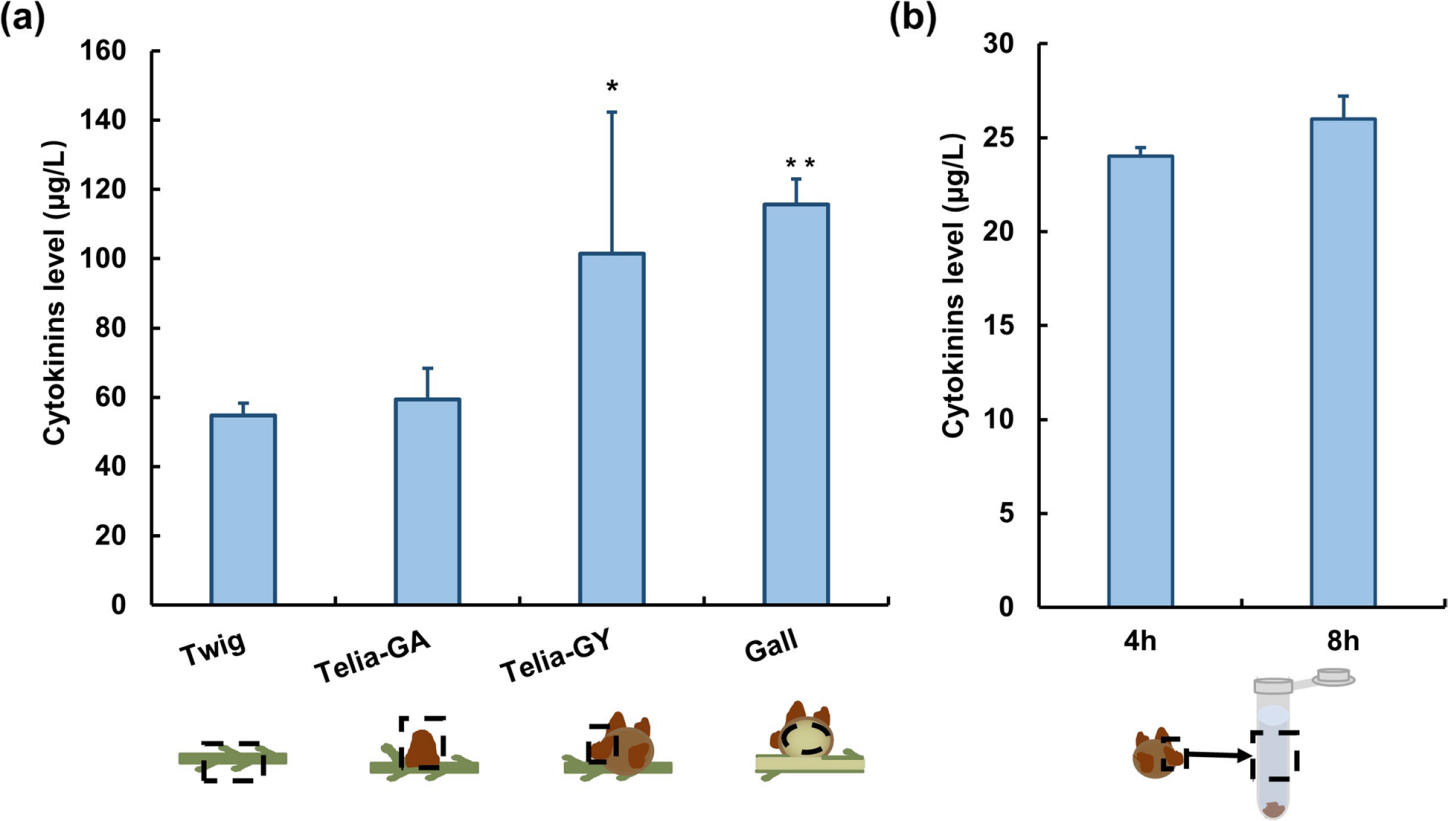
Levels of total cytokinins in juniper branches and telia of the two *Gymnosporangium* species. **a** Cytokinins content in the healthy juniper twig, *G. asiaticum* and *G. yamadae* and the galls. Telia-GA: the telia of *G. asiaticum*; Telia-GY: the telia of *G. yamadae*; Gall: *G. yamadae* induced gall tissues on juniper branches. **b** Cytokinins content in *G. yamadae* telia. 4 h, 8 h: the telia with the teliospores in supernatant of sterile water soaked after 4 h and 8 h. Total cytokinins were quantified by Plant Cytokinin (CTK) Enzyme-Linked Immunoassay Kit and Cytokinin (CTK) Enzyme-Linked Immunoassay Kit, respectively. The black boxes in the diagrammatic drawing below the abscissa show the specific sampling site of each sample. The samples to be tested were performed on microplate reader at OD_450nm_. Error bars are standard error (SE), *n* = 3

Gall tissue and the telia of *G. yamadae* contained substantially higher concentrations of CKs than healthy young branches, while concentrations of CKs in *G. asiaticum* telia were slightly higher than healthy young branches (Fig. 6a). To further test the ability of CKs production in *G. yamadae*, we examined the changes of CKs content during the teliospores germination. The result showed (Fig. 6b) that the CKs content was higher at 8 h than at 4 h during the teliospores germination.

**Fig. 7:**
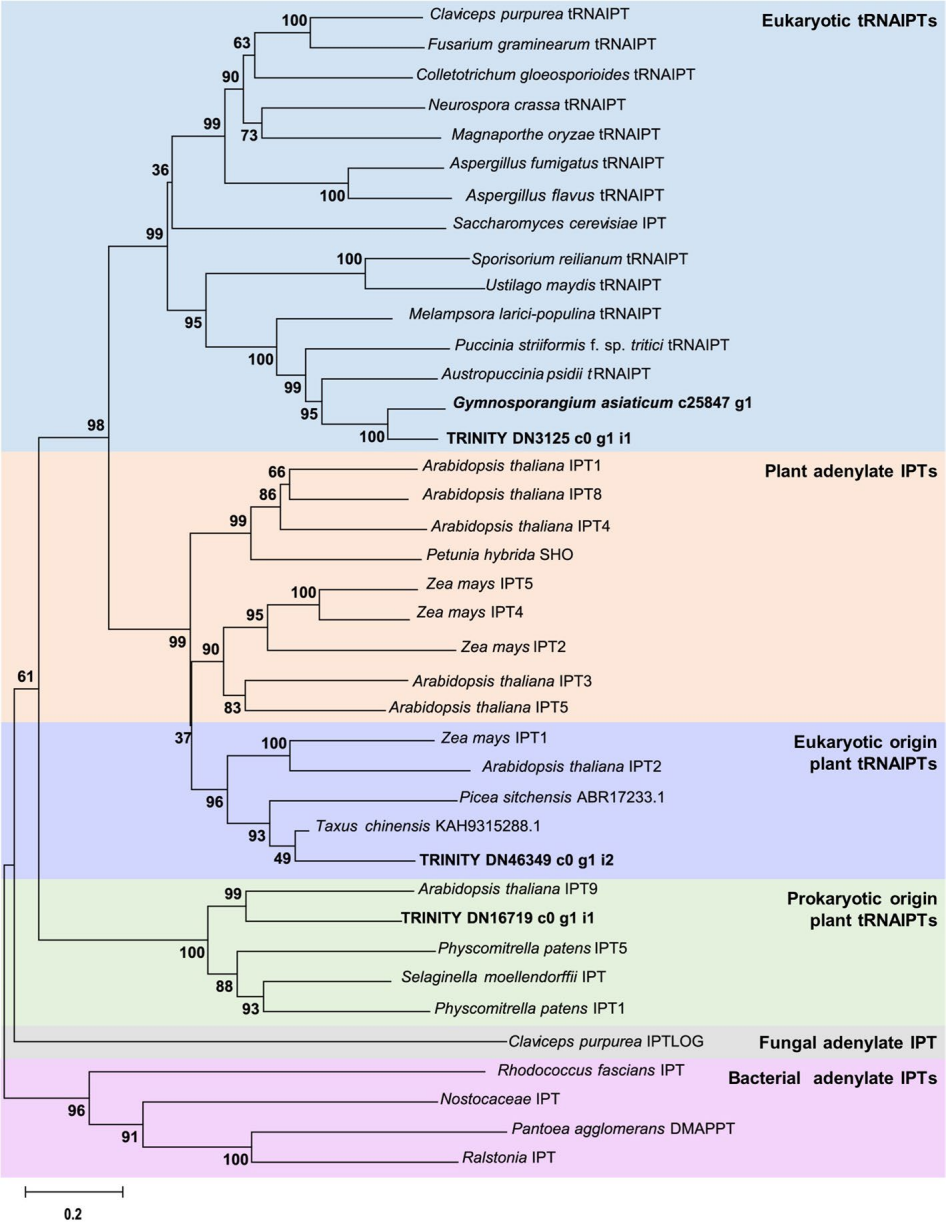
Phylogenetic relationship of tRNA-isopentenyltransferase proteins from a phylogenetically diverse group of organisms. The sequences were aligned using MAGA 6.0 using Neighbor-joining algorithm. Bootstrap values determined using 1000 replications, with the branch length indicating the expected amino acid changes per. tRNA-IPTs of *Gymnosporagium asiaticum*, *G. yamadae*, and juniper were highlighted using bold font

The features of CKs distribution in the gall of *G. yamadae* facilitate our study of the possibility that *G. yamadae*-derived CKs. We searched the KOG annotation results in our previously sequenced transcriptomes of *G. asiaticum* and *G. yamadae* teliospores and identified *c25847_g1* and *c18939_g3* annotated as “tRNA delta (2)-isopentenyl pyrophosphate transferase”. However, tRNA-IPT identified in this study was obtained from only one candidate, *TRINITY_DN3125_c0_g1_i1*, in juniper transcriptome database (Additional file 2: Table S7). We confirmed *c18939_g3* and *TRINITY_DN3125_c0_g1_i1* were one gene by protein sequence alignment, and identified *TRINITY_DN3125_c0_g1_i1* as *G. yamadae* tRNA-IPT (*GytRNA-IPT*), as well as *G. asiaticum* candidate tRNA-IPT (*c25847_g1*) and two juniper candidate tRNA-IPTs (*TRINITY_DN46394_c0_g1_i2* and *TRINITY_DN16719_c0_g1_i1*) were identified in this study to examine the relatedness to other tRNA-IPTs from a phylogenetically distinct group of organisms (Additional file 2: Table S7). An unrooted phylogenetic tree was constructed after trimming and aligning the tRNA-IPT sequences (Fig. 7). The phylogenetic tree was divided into six clusters, which were eukaryotic tRNA-IPTs, plant adenylate IPTs, eukaryotic origin plant tRNA-IPTs, prokaryotic origin plant tRNA-IPTs, fungal adenylate IPT and bacterial adenylate IPTs. In the phylogenetic tree, GytRNA-IPT was grouped with c25847_g1 in the cluster of eukaryotic tRNA-IPTs of plant pathogenic fungi. Alignment of GytRNA-IPT and c25847_g1 with other biotrophic fungal tRNA-IPTs, revealed conservation of ATG/GTP binding site, DMAPP binding site and a typical eukaryotic tRNA-IPTs zinc-finger domain in C-terminal (Additional file 1: Fig. S5). We noted that a chloroplast target sequence (18–54 amino acids) was identified within the N-terminal extension, and two nuclear localization signals (KKLWNEHVQSKRHRS, KRHRSASRPRKSYQHHKK) were predicted for the C-terminus of the protein.

**Fig. 8:**
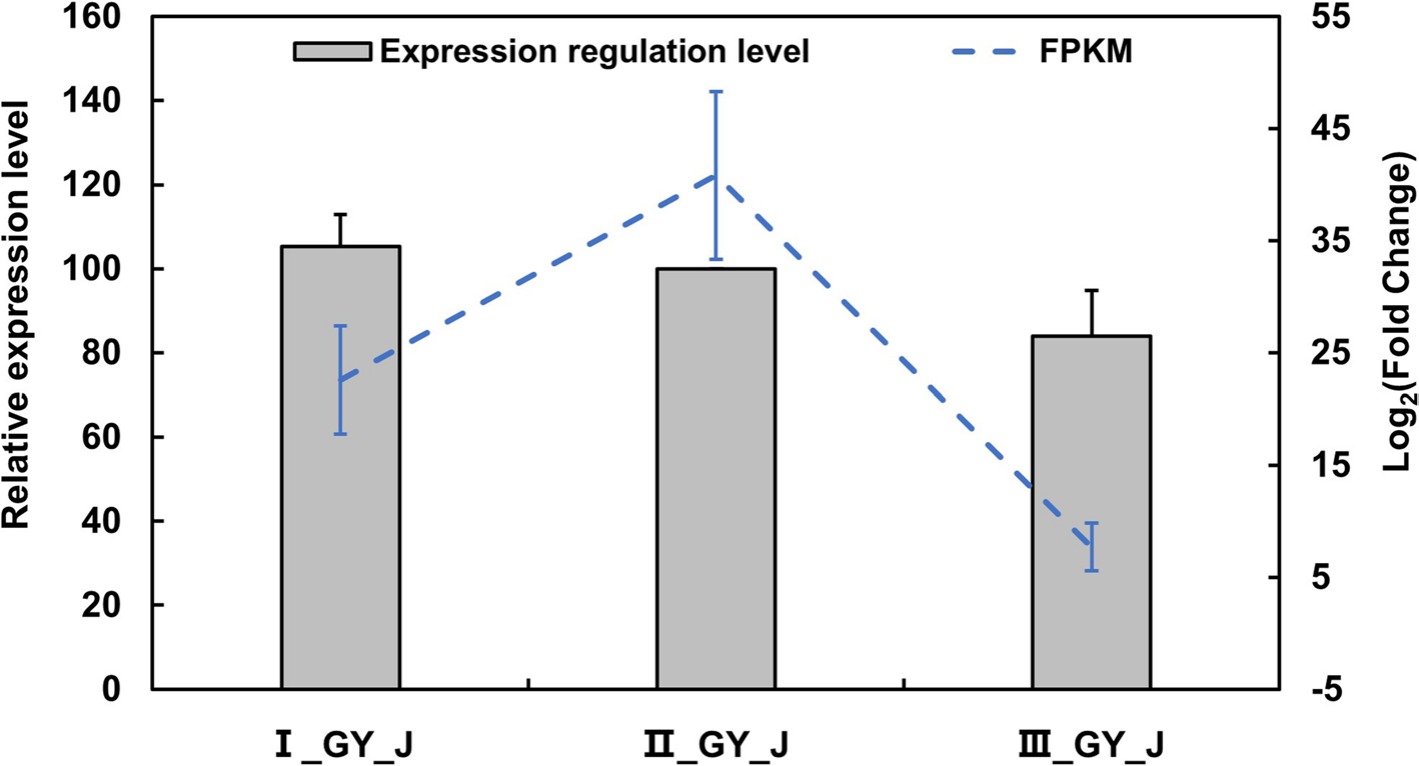
Expression profiles of *tRNA-isopentenyltransferase* (*GytRNA-IPT*) in different development stages of the gall. The bar graph displayed *GytRNA-IPT* relative expression levels by RT-qPCR with the normalization of *ubiquitin-conjugated enzyme E3*; error bars indicate standard error for three biological replicates are represented. Dotted lines represented FPKM value of the RNA-seq data; error bars indicate standard error, *n* = 3. The left y-axis represents relative expression level in RT-qPCR tests, the right y-axis represents log_2_Fold-Change in RNA-seq data, the x-axis represents three samples of the early stage (I_GY_J), the middle stage (II_GY_J) and the late stage (III_GY_J) of *Gymnosporagium yamadae*-induced gall development

The expression level of *GytRNA-IPT* was up-regulated in the middle stage (II_GY_J) relative to in the early stage (I_GY_J) and the late stage (III_GY_J) of gall development (Fig. 8). The RT-qPCR results of *GytRNA-IPT* were consistent with the FPKM values in the transcriptome data. These results indicated that the synthesis of CKs is stronger in gall tissues than in the branch tissues where galls are formed.

## Discussion

### The two *Gymnosporangium* species induced specific transcriptional responses during the biotrophic interactions with juniper

Generally, genes associated with photosynthesis and disease resistance in host plants are inhibited by rust pathogens during their infections, which are well known to be an indispensable infection strategy of obligate biotrophic pathogens [41–43]. In the present study, juniper genes related to photosynthesis and metabolism of terpenoids and polyketides, linolenic acid and α-linolenic acid in both *G. asiaticum* and *G. yamadae* infected tissues, which suggesting general responses of plant after pathogen infection [16, 23, 44]. However, up-regulated genes in *G. yamadae* induced gall tissues enriched in various pathways related to substance synthesis and metabolism, suggesting a more active metabolic activity in *G. yamadae* induced gall tissues than in *G. asiaticum* infected juniper tissues.

The plant responses to biotic stress including recognition of the initial signal from pathogen, followed by triggering a series transcriptional cascade and finally inducing the production of defense molecules [45, 46]. At the very late infection stage of *G. asiaticum* on juniper, downstream signal transduction and defense mechanism were extensively activated whereas bypassed initial signal recognition. However, major genes, particularly related to signal recognition, transduction and PR-proteins encoding were inhibited in *G. yamadae* induced gall tissues. It reflected that the differences of the two *Gymnosporangium* species infection strategies originally occurred at the molecular level of signal recognition in their interactions with juniper.

### Temporal transcriptional analysis revealed a *G. yamadae*-manipulated gene regulation pattern associated with gall development

Insects-induced leaf galls as resource sinks and shelters for these living organisms, for which exhibited wide range of gene expression changes, referring to the inhibition of genes related to photosynthesis and plant immune defense response and activation of genes related to nutrient metabolism [16, 17, 29, 47]. In the present study, genes associated with photosynthesis, chlorophyll synthesis and porphyrin and chlorophyll metabolism pathways were suppressed at the early and the late stages of the gall development [47]. Unexpectedly, a specific set of genes was significantly up-regulated at the middle stage (II_GY_J) and involved in light-harvesting and excitation transfer category (psaG, psbW and LHCB4), electron transporting (petF) and redox (petC), which may essentially constructed a cooperative framework of the two photosystems [48–50]. Our study revealed that photosynthesis-related genes were not continuously suppressed during gall development, and the enhanced photosynthetic activity may be responsible to produce more nutrients during the maturity of *G. yamadae* teliospores [27].

Genes involved in ‘glycine, serine and threonine metabolism’ pathway at the early stage (I_GY_J) of the gall development may suggest the nutrient reserves in gall tissues. More importantly, the increase of soluble sugar content might contribute to the elevated osmotic, thus increasing the water content in gall tissues [51]. Subsequently, along with *G. yamadae* teliospores development and maturity, vesicular transport-related genes and additional starch and sucrose synthesis-related genes were up-regulated at the middle stage (II_GY_J) and numbers of energy metabolism-related genes were up-regulated in the late stage (III_GY_J), which suggested a general function of galls as a carbohydrate source for the growth of *G. yamadae* teliospores. Correspondingly, rapid changes in the water and nutrient content in the gall lead to an increase in osmotic pressure, which induces the expression of ABA synthesis-related genes and an increase in ABA content, especially during the formation of large numbers of teliospores (II_GY_J) [33, 52]. Besides, genes involved in auxin signaling transduction pathway were also specifically up-regulated in II_GY_J, which possibly contributed to teliospores germination [20, 53, 54]. These results collectively reflected an autotrophic characteristic in *G. yamadae* induced gall tissues and indicated that the gall probably provide nutrients and water for teliospores of *G. yamadae* during the long-time development [29].

Many studies demonstrated that insects may have to suppress plant resistance systems during gall formation and development for better survival [16, 29]. However, we noted that a large number of plant defense response-related genes were activated during the middle stage (II_GY_J) of gall development. In addition, JA and SA signal transduction pathways were progressively activated at the middle stage (II_GY_J) and triggered defense-related response in juniper, which may be a result of the stronger interaction between *G. yamadae* and juniper along with the gall development and the teliospores maturation. Ultimately, the delayed activation of juniper defense responses at the late stage (III_GY_J) reflected a common infection strategy of biotrophic and hemibiotrophic plant pathogens [42, 55–57].

The main distinguishing features between other parasite-gall systems are the observation of gene expression profiles and the capture of dynamical changes in multiple developmental stages of gall, consistent with the phenotypic development of gall and the interaction between *G. yamadae* and juniper [29, 57, 58]. Furthermore, differences in the genetic regulation of host plant physiological responses, referring to secondary metabolism and stress defense mechanisms, maybe caused by phenotypic disparities [59–61]. Gymnosperms, especially conifers, are quite different from angiosperms in terms of leaf physical morphology and substance metabolism [61–64]. Consequently, the molecular mechanisms of fungi gall initiation and development remains an open question due to the lack of study on fungi-induced galls development and the difficulties in studying non-model plants [29].

### *G. yamadae*-derived cytokinins synthesized related with tRNA-IPT involved in the regulation of gall development

CKs plays an important role in the information and development of galls induced by insects and in the colonization and virulence of plant pathogenic fungi [20, 25, 38, 65]. While it is unclear whether there is rust-derived cis-zeatin. A recent study confirmed that cis-zeatin was detected in *G. juniper-virginianae* induced gall tissues as well as the wet telia, but not in control cedar branchlets [33]. To verify the effect of *G. yamadae* derived CKs on the induction of galls, we firstly examined the CKs content in juniper and *G. yamadae*, and found that galls and the telia contained significantly higher concentrations of CKs than the control sample (Fig. 6), consistent with the result in *G. juniper-virginianae*-galls system [33]. Considering the close relationship between *G. yamadae* and *G. juniper-virginianae* from a phylogenetic point of view [33], we proposed that cis-zeatin synthesized by the tRNA degradation pathway is responsible for a large number of CKs in *G. yamadae* consistent with demonstrating that the expression pattern of *GytRNA-IPT* gene was consistent with the developmental characteristics in *G. yamadae* induced galls. Although we were unable to verify direct regulation of the *tRNA-IPT* gene in the synthesis of CKs in *G. yamadae* due to the lack of genetic transformation in obligate biotrophic rust, the above results suggest that *G. yamadae*-derived CKs were essential in the gall formation and development. More importantly, *G. yamadae*-derived CKs in the galls may also play a role in nutrient mobilization and delaying senescence in juniper young branches, allowing the biotrophic *G. yamadae* to establish and grow in the host [24, 65].

We identified the orthologues in *G. yamadae* (GytRNA-IPT) from our published data which contains all the typical characteristics of eukaryotic tRNA-IPTs [66, 67] and shows a close phylogenetic relationship with *C. purpurea* tRNA-IPT and *U. maydis* tRNA-IPTs whose functions have been well understood, which suggested their similar functions [25, 38]. The derivation of two juniper candidate tRNA-IPTs (TRINITY_DN46394_c0_g1_i2 and TRINITY_DN16719_c0_g1_i1) from both eukaryotic origin plant tRNA-IPTs and prokaryotic origin plant tRNA-IPTs indicates a functional differentiation of juniper tRNA-IPTs. The phylogenetic tree also showed that tRNA-IPTs from obligated biotrophic fungi, such as *Austropuccinia psidii*, *Puccinia striiformis* f. sp. *tritici* and *Melampsora larici-populina* were clustered in eukaryotic tRNA-IPTs clade (Fig. 7). Further studies focusing on the functions of those *tRNA-IPT* genes would be instructive in understanding the role of fungal-derived CKs in plant-fungi interactions.

A surprising finding was that GytRNA-IPT contains a predicted chloroplast transit peptide within the N-terminal extension. So far, tRNA-IPTs with chloroplast transit peptide have only been found in *Arabidopsis* [68]. We confirmed that tRNA-IPT of *M. oryzae* also have a chloroplast transit peptide for N-terminus (Additional file 2: Table S7) whereas have no description in the previous study [39]. One possibility is that tRNA-IPT in fungi can be transferred to function in host plants, similar to effectors.

### Differentially utilizing of CKs providing a new insight into host selection mechanisms of rust fungi

Studies on the molecular mechanisms of rust-host plant specific interactions have shown that rust secretes effector proteins that largely determine host selection [15, 69, 70]. Nonetheless, the molecular mechanisms of rust-host plant interactions are mostly constrained from the perspective of effector proteins due to the lack of a genetic transformation system for rust. In contrast, in more plant-pathogen systems, secondary metabolites show a strong association with host specificity [71]. As for *Alternaria alternata*, a conditionally dispensable chromosome controls toxins production that has a contribution to host-specific pathogenicity [72]. At the genome level of *Suillus*, both secondary metabolites and pathways play a key role in enhancing host specificity by deactivating of reactive oxygen species [73]. Based on the current experimental data, we propose that *G. yamadae* induced galls determined by cZ-type CKs synthesized via GytRNA-IPT catalysis cannot be omitted in *G. yamadae*-juniper specific interactions. Interestingly, CKs are also accumulated in alternate host lobes without gall formation during the *G. yamadae*-apple interaction [33]. Combined with previous studies [28, 32], it is strongly suggested that there are other factors (e.g., effectors and small RNAs) in *G. yamadae*-juniper system that determine gall formation and development along with cZ-type CKs. The different infection characteristics of *G. yamadae* in juniper and apple leaves reflected the complicated mechanisms underlying the host-specific interactions of heteroecious rust.

*Gymnosporangium* species have a special host alternation pattern with its telial stage occurring on gymnosperms and aecial stage on angiosperms, which is contrary to most rusts parasitizing on gymnosperms during the aecial stage, such as *Cronartium ribicola* and *M. larici-populina* [8, 74, 75]. The majority of *Gymnosporangium* species have a narrow range of telial host selection restricted to only one or two genera, which usually results in different *Gymnosporangium* species parasitizing on the same tree, such as *G. asiaticum* and *G. yamadae* [4, 8, 13]. The two co-evolved *Gymnosporangium* species may exhibit strategies to avoid interspecific competition. Galls induced by *G. yamadae* on juniper branches ensure adequate nutrient and water during the long-time germination of the teliospores, thus providing more opportunities to transfer to alternative host. On the other hand, the adaptive trait of *G. yamadae* induced galls may be beneficial to avoid the competition of nutrients and water with *G. asiaticum* teliospores that also parasites on juniper. We hypothesized that the two *Gymnosporangium* species have evolved different infection strategies, i.e., different utilization of phytohormones, during their long evolutionary period, for the harmonious and common utilization of limited host resources. Although exactly how *G. yamadae* obtained the ability to induce the gall during the evolution remains unknown. Future work should emphasize the regulation of juniper cytokinin levels through pharmacological approaches, potentially disrupting teliospore formation as a strategy to manage rust disease.

## Conclusions

In this study, we explore gene regulatory changes in juniper following *G. asiaticum* and *G. yamadae* infection through comparative transcriptome analysis to further understand the development and host specificity of *Gymnosporangium* species symptoms. Temporal transcriptional analysis revealed the extensive reprogramming of gene expression in gall tissues induced by *G. yamadae*. More importantly, we proposed that *G. yamadae* derived CKs depended on *GytRNA-IPT* regulation is an essential factor in the gall formation, which reflects a conservative characteristic of biotrophic pathogenic fungi to use their own phytohormones for infection. The different infection strategies of *G. asiaticum* and *G. yamadae* remind us that the factors determining the host-specific interaction of rust fungus are not single and may relate to secondary metabolites such as phytohormones.

## Methods

### Juniper branch morphology and anatomy

The upper surface and longitudinal sections of *G. asiaticum* telial on juniper leaves and *G. yamadae* telial on juniper branches were photographed by a digital camera (Leica, M205FA). To illustrate the morphology and anatomy of the gall tissues, infected branches of *G. yamadae* at the middle stage of gall development, with full-grown gall and obvious teliospores, were utilized. Healthy juniper branches and the gall tissues were collected simultaneously from a single juniper tree. The branches or galls were cut into longitudinal sections by hand or into 0.2 mm thick sections using a freezing microtome (Leica CM1950) for cross-sections. Longitudinal sections of branch were visualized under a stereomicroscope (Leica, M205FA); cross-sections of healthy branch and gall tissues were photographed by light microscopes (Leica, DM3000 and Leica, DM2500).

### Experimental setup and sample collection

Juniper leaves and young branches infected by *G. asiaticum* and *G. yamadae*, including the early stage of gall development without the teliospores formation (I_GY_J), the middle stage with the teliospores break through the gall (II_GY_J) and the late stage of gall development with the matured teliospores (III_GA_J; III_GY_J), and healthy leaves and young branches (0_GY_J and 0_GA_J) as control were collected from Northwest A&F University, Yangling, Shaanxi and Haidian park, Beijing, China, between April and May 2021. All plant material were identified by Prof. Yingmei Liang. All samples were deposited in the Mycological Herbarium, Museum of Beijing Forestry University Mycologicum, Academiae (BJFC). Deposition number were: BJFC-R01700 (0_GA_J), BJFC-R02162 (III_GA_J), BJFC-R01692 (0_GY_J), BJFC-R02376 (I_GY_J)), BJFC-R02382 (II_GY_J), BJFC-R03656 (III_GY_J). Figure 1 and Additional file 1: Fig. S1 illustrate the experimental design. Sections of juniper tissues colonized by rust were collected and removed apparent fungal materials. Three biological replicates (i.e., J1, J2, J3) were harvested from each juniper, frozen separately in liquid nitrogen and stored at − 80 ℃ prior to RNA extraction.

### RNA extraction, RNA-sequencing and assembly

Total RNA was isolated from 100 mg of infected leaf and branch tissues using the RNA Easy Fast Plant Tissue Kit (Tiangen, Beijing) according to the manufacturer’s instructions. RNA quality and quantity were checked with a Qubit® RNA Assay Kit with a Qubit® 2.0 Fluorometer (Life Technologies, CA, USA). As shown in Additional file 1: Fig. S1, we constructed the cDNA libraries according to the standard protocols developed by Illumina (Illumina, San Diego, CA, USA) following the manufacturer’s recommendations. In each library, 150 bp paired-end reads were generated from Illunima NovaSeq 6000 platform (Illumina, San Diego, CA, U.S.A.). The raw sequenced reads were processed by FASTQ [76]. Next, the clean reads were used for de novo assembly using the Trinity software [77]. The kallisto software [78] was used to estimate the expression abundance of the assembled transcripts with default parameters. Further cluster analysis by using CD-HIT software package [79] retained only one of the transcripts whose similarity exceeded the threshold. Such sequences removed from redundancy based on similarity are defined as unigenes.

In order to assess the quality and homology of the transcriptome data, we mapped the unigenes of *J. chinensis* to the genomes of *Pinus lambertiana* (GCA_001447015.2_Sugar_pine_JHU_assembly_genomic.fna), *Pinus taeda* (GCA_000404065.3_Ptaeda2.0_genomic.fna), *Picea abies* (GCA_900067695.1_Pabies01_genomic.fna), *Picea glauca* (GCA_000411955.6_PG29_v5_genomic.fna) and *Gnetum montanum* (Gnetum.final.fa) through BLASTn and BLASTp [80] with default parameters in NCBI. The unigenes of *G. asiaticum* and *G. yamadae* were annotated as described in the previous study [4]. The unigenes that without comparison results were further annotated by a subset of plants and fungi from the NR database (NCBI non-redundant protein sequences). All unigenes that can be aligned were used to generate the transcriptomes of *J. chinensis*, *G. asiaticum* and *G. yamadae* (Additional file 1: Fig. S1).

TransDecoder (https://github.com/TransDecoder/TransDecoder/wiki) was used to predict the coding sequences (CDS) for all unigenes. Gene function was annotated in KO (KEGG Ortholog database), GO (Gene Ontology), Pfam (Protein family), and NR databases by EggNOG-emapper [81] and in Swiss-Prot (A manually annotated and reviewed protein sequence database), KOG/COG (Clusters of Orthologous Groups of proteins) databases by diamond [82], respectively.

### Gene expression analysis

The FPKM expression levels and counts for all unigenes were estimated in each replicate by RSEM [83]. Principal component analysis (PCA) was performed from read counts via the plotPCA function in R package [84]. The similarity between samples at the expression levels was visualized by calculating the Pearson correlation coefficient between samples. Different expression genes (DEGs) was performed by DEseq2 [85] and unigenes with a significant *p*-value (< 0.05) and more than a twofold change in transcript level were deemed as differentially expressed. Volcano plots of DEGs and heatmaps of gene expression profiles were generated using TBtools software [86]. To derive expression patterns of genes in the different gall development stages, unigenes expressed at all stages were selected across all the stages with *p*-value < 0.05.

### Functional analysis of DEGs

The annotated DEGs were used in functional enrichment analysis. Kyoto Encyclopedia of Genes and Genomes (KEGG) pathways enrichment analysis [87] and GO category annotation of significant DEGs were performed by clusterProfiler [84] with adjusted *p*-value < 0.05, and the TBtools software [86] was used to test the statistical enrichment of differentially expressed genes. In addition, significant DEGs were classified to functional categories using the pathway analysis program MapMan [88] with default parameters.

### CKs extraction and quantitative analyses

Total cytokinins were extracted from 100 mg of healthy branches, *G. yamadae* infected young branches (galls) and the telial of *G. asiaticum* and *G. yamadae*. Measured teliospores were artificially germinated by immersing them in distilled water [89]. Then the supernatant was took at the middle (4 h) and late (8 h) time stages to measure the CKs content. Briefly, all samples were ground in liquid nitrogen, and then lysed by PBS and RIPA buffer, respectively. The obtained 1 mg/mL homogenates were used for CKs content determination. Afterward, 10µL supernatant were used for quantitative analyses by using Plant Cytokinin (CTK) Enzyme-Linked Immunoassay Kit (WuHan Saipei Biotechnology, WuHan) to detect plant samples and using Cytokinin (CTK) Enzyme-Linked Immunoassay Kit (WuHan Saipei Biotechnology, WuHan) to detect teliospores samples performed in Multiskan Go (Thermo scientific, USA), according to the manufacturer’s instructions.

### Identification of potential cytokinins biosynthetic genes in *G. asiaticum* and* G. yamadae* and phylogeny analyses

Based on the previous published data [4], potential tRNA-isopentenyl transferase genes were identified in telial transcriptomes of *G. asiaticum* and *G. yamadae*. TBtools Reciprocal BLAST was then conducted to search for homologous genes in the transcriptome of this study. Combined the results from previous studies [25, 66], we added 1 tRNA-IPT from *Cholletotrichum gloeosporioides* (hemitropic fungi) and 3 tRNA-IPTs from rust (biotrophic fungi) through a BLASTp search [80] in NCBI. There were 39 tRNA-IPT protein sequences listed in Additional file 2: Table S7 and the identified tRNA-IPT orthologues of *G. asiaticum* and *G. yamadae* were aligned using MAGA 6.0 using Neighbor-joining algorithm. Bootstrap values were determined using 1000 replications. Protein sequences were aligned by multiple alignment with SnapGene 6.1 and visualized using GeneDoc [90]. Localization signals of *G. yamadae* tRNA-IPT protein were predicted by using LOCALIZER [91].

### RT-qPCR validation

RT-qPCR analysis was conducted to validate genes expression profiles between infected leaf samples and healthy leaf samples. Primers were designed by Primer 3 [92] and synthesized by RuiBioTech (Beijing, China). Primer information is presented in Additional file 7: Table S7. The RT-qPCR was performed in CFX96™ SYBR® Real-TimePCR System (Bio-Rad Laboratories, Hercules, CA, USA), as described previously [93]. Transcript expression levels were normalized with *ubiquitin-conjugated enzyme E3* [94]. Data logging and the determination of Cq values were done using Bio-Rad CFX Manager 2.1 (Bio-Rad Laboratories, Hercules, CA, USA).

## Supplementary Information

**Additional file 1: Figure S1.** The pipeline of RNA-seq and bioinformatic analysis used in the study. The plant unigenes were mapped to five publicly available databases of *Pinus lambertiana*, *P.s taeda*, *Picea abies*, *P. gluca*, *Gnetum montanum* in National Center for Biotechnology Information. The rust fungi unigenes were annotated as described in the methods. **Figure S2.** The proportional distribution by species of *Juniperus chinensis*** (a)** and *Gymnosporangium* species **(b)** unigenes with homology in NR database. **Figure S3.** Assessment of RNA-seq data reproducibility. **(a)**, Principal component analysis based on gene expression level of healthy leaves and branches (0_GA_J and 0_GY_J), *Gymnosporangium asiaticum* infected leaves with the the matured teliospores (III_GA_J), *G. yamadae* infected branch tissues at the early (I_GY_J), middle (II_GY_J) and late stage (III_GY_J) of the gall development, showing the clear separation of the six tested samples and the proximity of biological replicates. **(b**), Sample correlation matrix of replicates of healthy leaves and branches (0_GA_J and 0_GY_J), *G. asiaticum* infected leaves with the the matured teliospores (III_GA_J), *G. yamadae* infected branch tissues at the early (I_GY_J), middle (II_GY_J) and late stage (III_GY_J) of the gall development based on gene expression. **Figure S4.** Statistics of annotated different expression genes. Venn diagram showing the number of up- and down-regulated genes (|log_2_Fold-Change| > 1, *p*-value < 0.05) in III_GA _J and III_GY_J samples compared with the control, respectively. Type_a and Type_b refer to juniper unigenes regulated reversely in III_GA _J and III_GY_J samples. **Figure S5.** Amino acid sequence alignment of tRNA-IPT homologs. The tRNA-IPTs from *Saccharomyces cerevisiae* (NP_014917.3), *Claviceps purpurea* (CCE29200.1), *Sporisorium reilianum* (CBQ67583.1), *Ustilago maydis* (XP_011386632.1), *Gymnosporangium asiaticum *(c25847_g1) and *Gymnosporangium yamadae* (TRINITY_DN3125 c0_gI_i1). The dark to light colors indicate amino acids conservation from high to low. Black lines indicate ATG/GTP binding site, DMAPP binding site and zinc-finger motif; the two putative nuclear localization signals of GytRNA-IPT (TRINITY_DN3125 c0_gI_i1) and GatRNA-IPT (c25847_g1) are highlighted by green and red boxes; black box indicate chloroplast localization signal of GytRNA-IPT.

**Additional file 2: Table S1.** The statistics of unigene classification. Unigenes assigned with “juniper” species were determined as juniper unigenes, whie assigned with “fungi” species were determined as the two *Gymnosporangium* spp. unigenes. **Table S2.** Functional annotation of *Juniperus chinensis* and the *Gymnosporangium* spp. unigenes in selected six public databases (Nr, KEGG, GO, Swissprot, KOG, PFAM). **Table S3.** Differentially expressed genes in III_GA, I_GY, II_GY and III_GY. **Table S4.** Swissport annotations of Type_a and Type_b DEGs in the Venn diagram. **Table S5.** MapMan annotations of different expression genes (|log_2_Fold-Change| ≥ 5, *p* <0.01) in III_GA and III_GY samples. **Table S6.** FPKM and KO number of photosynthesis, sugar metabolism, plant hormone and defense-related genes in I_GY, II_GY and III_GY samples. **Table S7.** The tRNA-isopentenyltransferase proteins used for sequence alignment and phylogenetic tree creation.

## Data Availability

The data that support the findings of this study are available from the corresponding author upon reasonable request. RNA-Seq raw data were obtained from the Sequence Read Archive (https://www.ncbi.nlm.nih.gov/bioproject/) under the accession numbers SRR23018171-SRR23018188.

## Abbreviations

CKs: Cytokinins
cZ: Cis-zeatin
DEGs: Differentially expressed genes
FPKM: Fragment per kilobase per million mapped reads
GO: Gene Ontology
0_GA_J and 0_GY_J: Juniper healthy branch tissues
IAA: Indole-3-acetic acid
I_GY_J: Juniper branch tissues infected by *G. yamadae* at the early stage of gall development without the teliospores formation
II_GY_J: Juniper branch tissues infected by *G. yamadae* the middle stage with the teliospores break through the gall
III_GY_J: Juniper branch tissues infected by *G. yamadae* the late stage of gall development with the matured teliospores
III_GA_J: Juniper branch tissues infected by *G. asiaticum* at the stage with the matured teliospores
iP: Isopentenyladenine
IPTs: Isopentenyltransferases
KEGG: Kyoto Encyclopaedia of Genes and Genomes
KOG: Eukaryotic orthologue
PCA: Principal component analysis
Pfam: Protein family
tRNA-IPT: tRNA-isopentenyltransferase
tZ: Trans-zeatin
