# Supplementary figures and images for: Comparative transcriptome analysis of juniper branches infected by Gymnosporangium spp. highlights their different infection strategies associated with cytokinins
Source: BMC Genomics. 2023 Apr 5;24:173. doi: 10.1186/s12864-023-09276-7 (PMC10077639; doi:10.1186/s12864-023-09276-7)

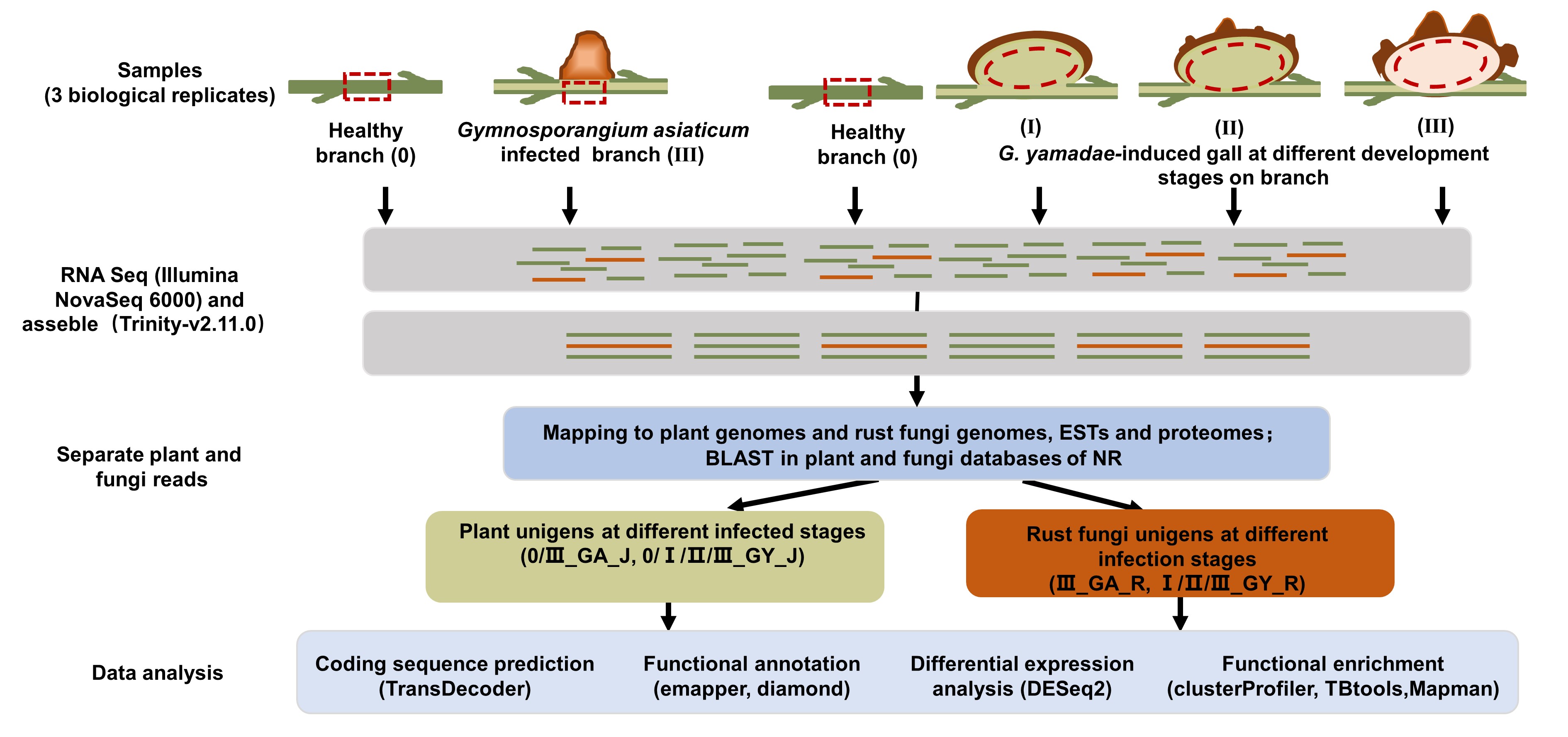

Supplement: Supplementary file 1 — Additional file 1: Figure S1. The pipeline of RNA-seq and bioinformatic analysis used in the study. The plant unigenes were mapped to five publicly available databases of Pinus lambertiana, P.s taeda, Picea abies, P. gluca, Gnetum montanum in National Center for Biotechnology Information. The rust fungi unigenes were annotated as described in the methods. Figure S2. The proportional distribution by species of Juniperus chinensis (a) and Gymnosporangium species (b) unigenes with homology in NR database. Figure S3. Assessment of RNA-seq data reproducibility. (a), Principal component analysis based on gene expression level of healthy leaves and branches (0_GA_J and 0_GY_J), Gymnosporangium asiaticum infected leaves with the the matured teliospores (III_GA_J), G. yamadae infected branch tissues at the early (I_GY_J), middle (II_GY_J) and late stage (III_GY_J) of the gall development, showing the clear separation of the six tested samples and the proximity of biological replicates. (b), Sample correlation matrix of replicates of healthy leaves and branches (0_GA_J and 0_GY_J), G. asiaticum infected leaves with the the matured teliospores (III_GA_J), G. yamadae infected branch tissues at the early (I_GY_J), middle (II_GY_J) and late stage (III_GY_J) of the gall development based on gene expression. Figure S4. Statistics of annotated different expression genes. Venn diagram showing the number of up- and down-regulated genes (|log2Fold-Change| > 1, p-value < 0.05) in III_GA _J and III_GY_J samples compared with the control, respectively. Type_a and Type_b refer to juniper unigenes regulated reversely in III_GA _J and III_GY_J samples. Figure S5. Amino acid sequence alignment of tRNA-IPT homologs. The tRNA-IPTs from Saccharomyces cerevisiae (NP_014917.3), Claviceps purpurea (CCE29200.1), Sporisorium reilianum (CBQ67583.1), Ustilago maydis (XP_011386632.1), Gymnosporangium asiaticum (c25847_g1) and Gymnosporangium yamadae (TRINITY_DN3125 c0_gI_i1). The dark to light colors in [file 12864_2023_9276_MOESM1_ESM.zip › Additional files1Figure S1.jpg]

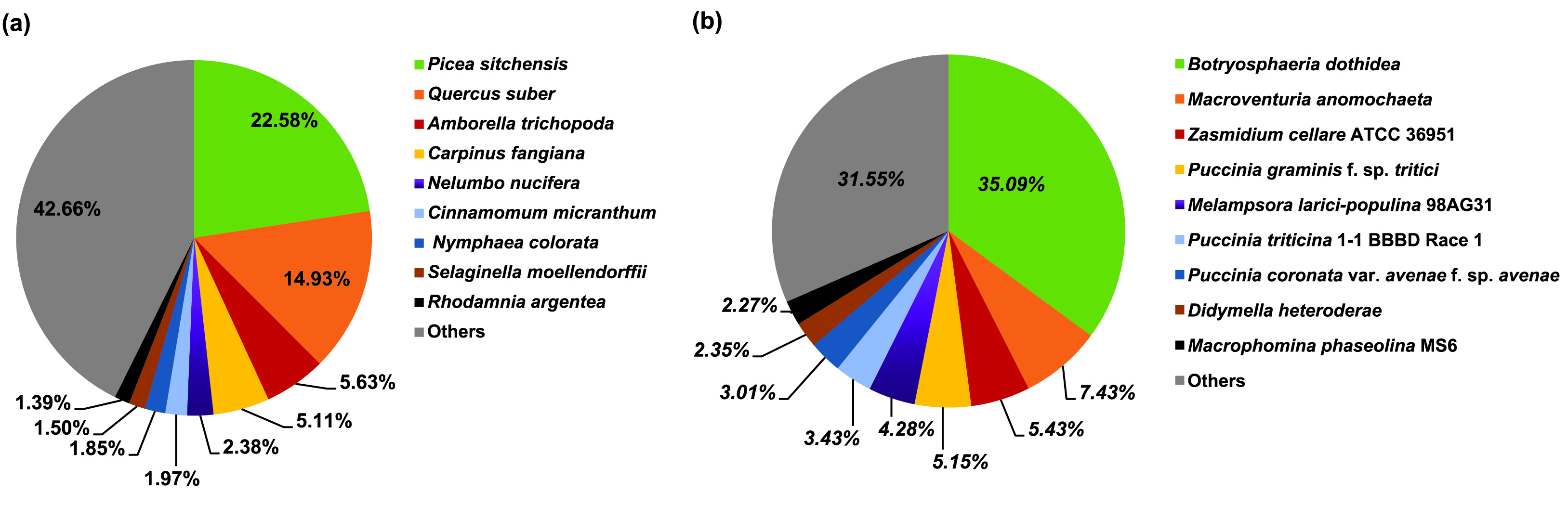

Supplement: Supplementary file 1 — Additional file 1: Figure S1. The pipeline of RNA-seq and bioinformatic analysis used in the study. The plant unigenes were mapped to five publicly available databases of Pinus lambertiana, P.s taeda, Picea abies, P. gluca, Gnetum montanum in National Center for Biotechnology Information. The rust fungi unigenes were annotated as described in the methods. Figure S2. The proportional distribution by species of Juniperus chinensis (a) and Gymnosporangium species (b) unigenes with homology in NR database. Figure S3. Assessment of RNA-seq data reproducibility. (a), Principal component analysis based on gene expression level of healthy leaves and branches (0_GA_J and 0_GY_J), Gymnosporangium asiaticum infected leaves with the the matured teliospores (III_GA_J), G. yamadae infected branch tissues at the early (I_GY_J), middle (II_GY_J) and late stage (III_GY_J) of the gall development, showing the clear separation of the six tested samples and the proximity of biological replicates. (b), Sample correlation matrix of replicates of healthy leaves and branches (0_GA_J and 0_GY_J), G. asiaticum infected leaves with the the matured teliospores (III_GA_J), G. yamadae infected branch tissues at the early (I_GY_J), middle (II_GY_J) and late stage (III_GY_J) of the gall development based on gene expression. Figure S4. Statistics of annotated different expression genes. Venn diagram showing the number of up- and down-regulated genes (|log2Fold-Change| > 1, p-value < 0.05) in III_GA _J and III_GY_J samples compared with the control, respectively. Type_a and Type_b refer to juniper unigenes regulated reversely in III_GA _J and III_GY_J samples. Figure S5. Amino acid sequence alignment of tRNA-IPT homologs. The tRNA-IPTs from Saccharomyces cerevisiae (NP_014917.3), Claviceps purpurea (CCE29200.1), Sporisorium reilianum (CBQ67583.1), Ustilago maydis (XP_011386632.1), Gymnosporangium asiaticum (c25847_g1) and Gymnosporangium yamadae (TRINITY_DN3125 c0_gI_i1). The dark to light colors in [file 12864_2023_9276_MOESM1_ESM.zip › Additional files1Figure S2.jpg]

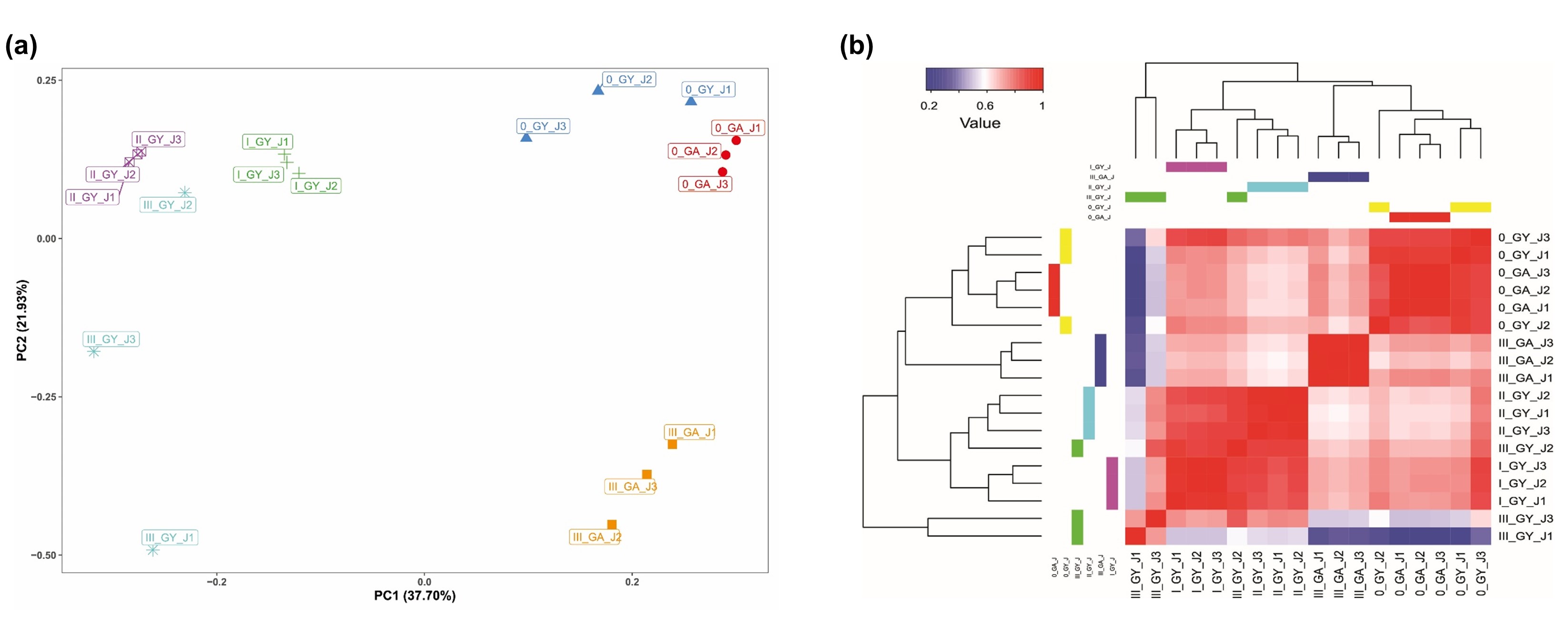

Supplement: Supplementary file 1 — Additional file 1: Figure S1. The pipeline of RNA-seq and bioinformatic analysis used in the study. The plant unigenes were mapped to five publicly available databases of Pinus lambertiana, P.s taeda, Picea abies, P. gluca, Gnetum montanum in National Center for Biotechnology Information. The rust fungi unigenes were annotated as described in the methods. Figure S2. The proportional distribution by species of Juniperus chinensis (a) and Gymnosporangium species (b) unigenes with homology in NR database. Figure S3. Assessment of RNA-seq data reproducibility. (a), Principal component analysis based on gene expression level of healthy leaves and branches (0_GA_J and 0_GY_J), Gymnosporangium asiaticum infected leaves with the the matured teliospores (III_GA_J), G. yamadae infected branch tissues at the early (I_GY_J), middle (II_GY_J) and late stage (III_GY_J) of the gall development, showing the clear separation of the six tested samples and the proximity of biological replicates. (b), Sample correlation matrix of replicates of healthy leaves and branches (0_GA_J and 0_GY_J), G. asiaticum infected leaves with the the matured teliospores (III_GA_J), G. yamadae infected branch tissues at the early (I_GY_J), middle (II_GY_J) and late stage (III_GY_J) of the gall development based on gene expression. Figure S4. Statistics of annotated different expression genes. Venn diagram showing the number of up- and down-regulated genes (|log2Fold-Change| > 1, p-value < 0.05) in III_GA _J and III_GY_J samples compared with the control, respectively. Type_a and Type_b refer to juniper unigenes regulated reversely in III_GA _J and III_GY_J samples. Figure S5. Amino acid sequence alignment of tRNA-IPT homologs. The tRNA-IPTs from Saccharomyces cerevisiae (NP_014917.3), Claviceps purpurea (CCE29200.1), Sporisorium reilianum (CBQ67583.1), Ustilago maydis (XP_011386632.1), Gymnosporangium asiaticum (c25847_g1) and Gymnosporangium yamadae (TRINITY_DN3125 c0_gI_i1). The dark to light colors in [file 12864_2023_9276_MOESM1_ESM.zip › Additional files1Figure S3.jpg]

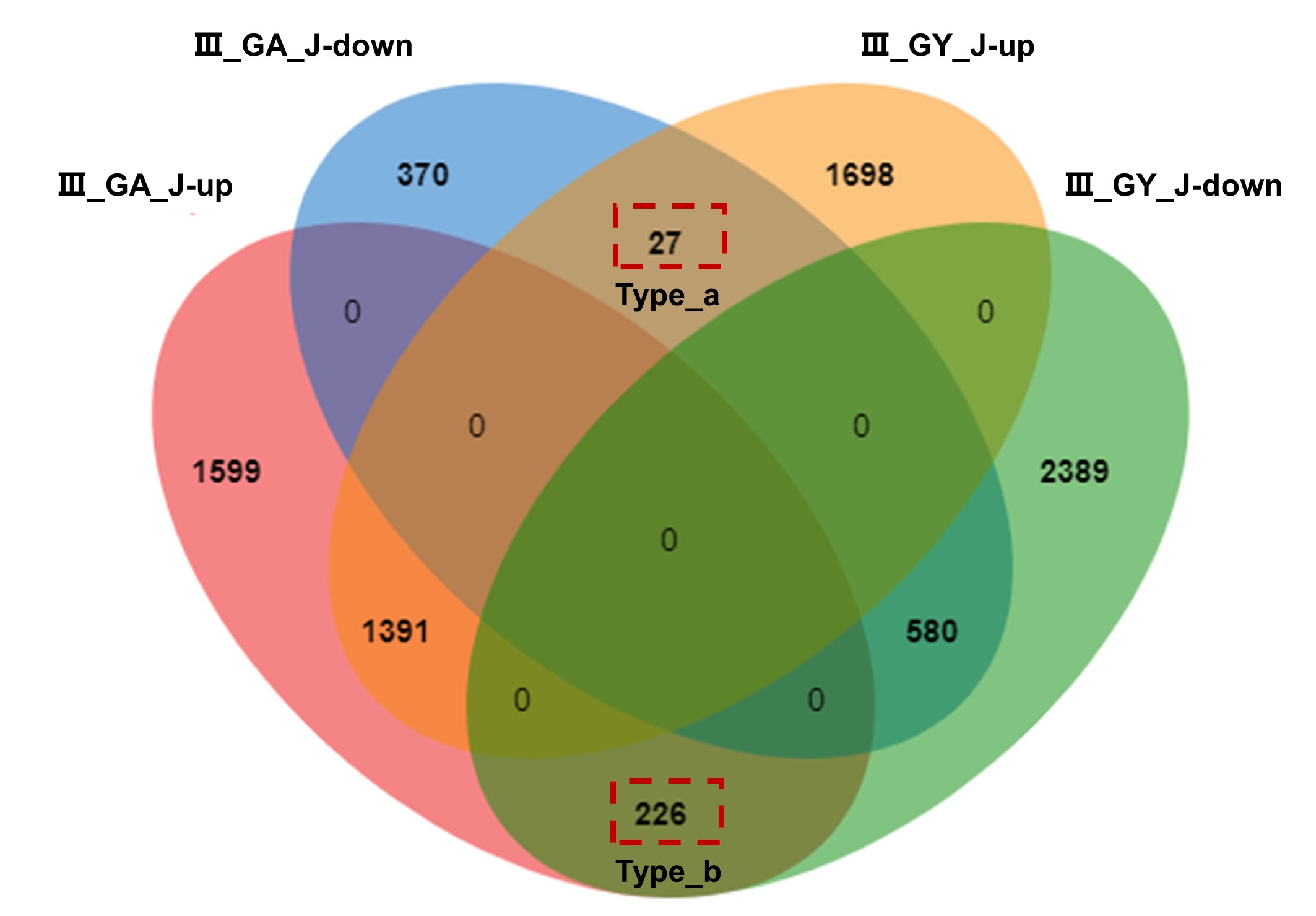

Supplement: Supplementary file 1 — Additional file 1: Figure S1. The pipeline of RNA-seq and bioinformatic analysis used in the study. The plant unigenes were mapped to five publicly available databases of Pinus lambertiana, P.s taeda, Picea abies, P. gluca, Gnetum montanum in National Center for Biotechnology Information. The rust fungi unigenes were annotated as described in the methods. Figure S2. The proportional distribution by species of Juniperus chinensis (a) and Gymnosporangium species (b) unigenes with homology in NR database. Figure S3. Assessment of RNA-seq data reproducibility. (a), Principal component analysis based on gene expression level of healthy leaves and branches (0_GA_J and 0_GY_J), Gymnosporangium asiaticum infected leaves with the the matured teliospores (III_GA_J), G. yamadae infected branch tissues at the early (I_GY_J), middle (II_GY_J) and late stage (III_GY_J) of the gall development, showing the clear separation of the six tested samples and the proximity of biological replicates. (b), Sample correlation matrix of replicates of healthy leaves and branches (0_GA_J and 0_GY_J), G. asiaticum infected leaves with the the matured teliospores (III_GA_J), G. yamadae infected branch tissues at the early (I_GY_J), middle (II_GY_J) and late stage (III_GY_J) of the gall development based on gene expression. Figure S4. Statistics of annotated different expression genes. Venn diagram showing the number of up- and down-regulated genes (|log2Fold-Change| > 1, p-value < 0.05) in III_GA _J and III_GY_J samples compared with the control, respectively. Type_a and Type_b refer to juniper unigenes regulated reversely in III_GA _J and III_GY_J samples. Figure S5. Amino acid sequence alignment of tRNA-IPT homologs. The tRNA-IPTs from Saccharomyces cerevisiae (NP_014917.3), Claviceps purpurea (CCE29200.1), Sporisorium reilianum (CBQ67583.1), Ustilago maydis (XP_011386632.1), Gymnosporangium asiaticum (c25847_g1) and Gymnosporangium yamadae (TRINITY_DN3125 c0_gI_i1). The dark to light colors in [file 12864_2023_9276_MOESM1_ESM.zip › Additional files1Figure S4.jpg]

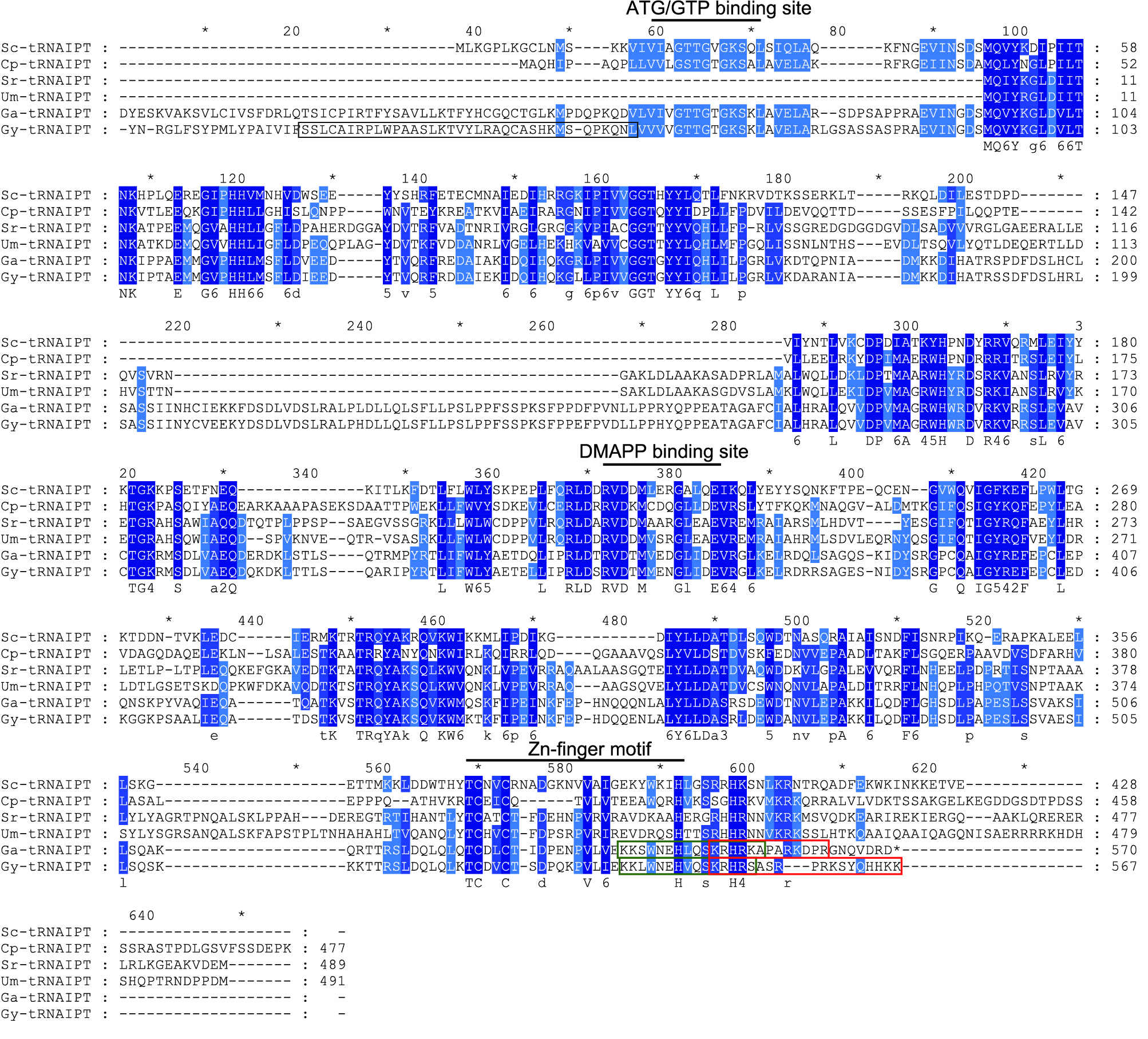

Supplement: Supplementary file 1 — Additional file 1: Figure S1. The pipeline of RNA-seq and bioinformatic analysis used in the study. The plant unigenes were mapped to five publicly available databases of Pinus lambertiana, P.s taeda, Picea abies, P. gluca, Gnetum montanum in National Center for Biotechnology Information. The rust fungi unigenes were annotated as described in the methods. Figure S2. The proportional distribution by species of Juniperus chinensis (a) and Gymnosporangium species (b) unigenes with homology in NR database. Figure S3. Assessment of RNA-seq data reproducibility. (a), Principal component analysis based on gene expression level of healthy leaves and branches (0_GA_J and 0_GY_J), Gymnosporangium asiaticum infected leaves with the the matured teliospores (III_GA_J), G. yamadae infected branch tissues at the early (I_GY_J), middle (II_GY_J) and late stage (III_GY_J) of the gall development, showing the clear separation of the six tested samples and the proximity of biological replicates. (b), Sample correlation matrix of replicates of healthy leaves and branches (0_GA_J and 0_GY_J), G. asiaticum infected leaves with the the matured teliospores (III_GA_J), G. yamadae infected branch tissues at the early (I_GY_J), middle (II_GY_J) and late stage (III_GY_J) of the gall development based on gene expression. Figure S4. Statistics of annotated different expression genes. Venn diagram showing the number of up- and down-regulated genes (|log2Fold-Change| > 1, p-value < 0.05) in III_GA _J and III_GY_J samples compared with the control, respectively. Type_a and Type_b refer to juniper unigenes regulated reversely in III_GA _J and III_GY_J samples. Figure S5. Amino acid sequence alignment of tRNA-IPT homologs. The tRNA-IPTs from Saccharomyces cerevisiae (NP_014917.3), Claviceps purpurea (CCE29200.1), Sporisorium reilianum (CBQ67583.1), Ustilago maydis (XP_011386632.1), Gymnosporangium asiaticum (c25847_g1) and Gymnosporangium yamadae (TRINITY_DN3125 c0_gI_i1). The dark to light colors in [file 12864_2023_9276_MOESM1_ESM.zip › Additional files1Figure S5.jpg]
