# Supplementary material for: Comparative transcriptome analysis of juniper branches infected by Gymnosporangium spp. highlights their different infection strategies associated with cytokinins
Source: BMC Genomics. 2023 Apr 5;24:173. doi: 10.1186/s12864-023-09276-7 (PMC10077639; doi:10.1186/s12864-023-09276-7)
Supplement: Supplementary file 2 — Additional file 2: Table S1. The statistics of unigene classification. Unigenes assigned with “juniper” species were determined as juniper unigenes, whie assigned with “fungi” species were determined as the two Gymnosporangium spp. unigenes. Table S2. Functional annotation of Juniperus chinensis and the Gymnosporangium spp. unigenes in selected six public databases (Nr, KEGG, GO, Swissprot, KOG, PFAM). Table S3. Differentially expressed genes in III_GA, I_GY, II_GY and III_GY. Table S4. Swissport annotations of Type_a and Type_b DEGs in the Venn diagram. Table S5. MapMan annotations of different expression genes (|log2Fold-Change| ≥ 5, p <0.01) in III_GA and III_GY samples. Table S6. FPKM and KO number of photosynthesis, sugar metabolism, plant hormone and defense-related genes in I_GY, II_GY and III_GY samples. Table S7. The tRNA-isopentenyltransferase proteins used for sequence alignment and phylogenetic tree creation. [file 12864_2023_9276_MOESM2_ESM.zip › Additional files2 Table S2.docx]

|  | *Juniperus chinensis* L. | *Gymnosporangium* spp. |
| --- | --- | --- |
| Annotated in NR | 29,039 (71.56%) | 25,514 (93.89%) |
| Annotated in KEGG | 17,221(42.44%) | 12,524 (46.09%) |
| Annotated in GO | 16,778 (42.44%) | 11,243 (46.09%) |
| Annotated in SwissProt | 23,682 (58.36%) | 14,742 (54.25%) |
| Annotated in KOG | 17,523 (43.18%) | 10,949 (40.29%) |
| Annotated in PFAM | 29,830 (73.51%) | 21,290 (78.35%) |
| Annotated in all Databases | 9,065 (22.34%) | 6,975 (25.67%) |
| Annotated in at least one Database | 32,200 (79.35%) | 2,5623 (94.30%) |
| Total unigens | 40,581 (100%) | 27,173 (100%) |

Table S2. Functional annotation of *Juniperus chinensis* and the *Gymnosporangium* spp. unigenes in selected public databases
